# Memoirs on the Nervous System

**Published:** 1838-04

**Authors:** 


### Art. XIII.

1. *Memoirs on the Nervous System.* By Marshall Hall, m.d. f.r.s. l. & e. &c.—*London*, 1837. 4to. pp. 113.

2. *Observations on the Structure and Functions of the Spinal Cord.* By R. D. Grainger, Lecturer on Anatomy and Physiology.—*London*, 1837. 8vo. pp. 159.

3. *Powers of the Roots of the Nerves, in Health and in Disease; likewise on Magnetic Sleep.* By Herbert Mayo, f.r.s., Senior Surgeon to the Middlesex Hospital.—*London*, 1837. 8vo. pp. 39.

Before giving any detailed account of the works whose titles head this article, we think it proper to sketch, as briefly but comprehensively as possible, what we may regard as the present state of our knowledge of the general structure and functions of the nervous system; and in this summary we shall freely avail ourselves of the materials which their respective authors have contributed. When any important modifications in received views demand introduction, it seems peculiarly desirable that they should be examined, not only as to their separate validity, but with regard to their correspondence with what was formerly known or understood; for many who have devoted their lives to the pursuit of truth naturally become wedded to their habitual trains of thought; and, when a novelty has been announced, are more apt to test its merits by its conformity with their opinions than to examine how far it may really accord with the facts upon which those opinions have been formed.

No physiologist has any doubt that one office of the nervous system is to bring the conscious mind (using this term in its extended sense, to include the psychical endowments of animals in general,) into relation with the external world; by informing it, through the medium of the organs of sensation, of the changes which material objects undergo, and enabling it to act by its appropriate organs upon other beings, animate and inanimate: and that it is also concerned in the production of many changes in the system of the individual, immediately connected with the maintenance of organic life. The enquiry into the mode and degree in which the nervous system ministers to these purposes involves a great variety of questions, some of which we shall not at present discuss. Our immediate object is to determine the extent to which purely *mental* acts are concerned in the production of bodily changes, and through what instruments their influence is communicated; and we shall defer until the close of this enquiry what we have to say of the respective merits of the numerous individuals for whom a claim to rank as discoverers of important truths in this field of enquiry, has been raised by themselves or others.

We have remarked in a former article that nothing is likely to be known of the functional alterations in the state of the nervous system, since these are of a kind imperceptible to our senses, even when aided by various means of rendering them more apparent. The microscope and the galvanometer have alike proved useless in the most skilful hands. We know, or rather we have the means of knowing, as much of *nervous influence* as of electricity or gravity: of the existence of any of these as distinct entities we may reasonably entertain doubts; and we only take cognizance of them as properties of matter in particular forms. No researches, therefore, into the functions of the nervous system can ever unveil more than the *mechanism* (if we may use the expression) by which impressions are propagated through its fibres, and the properties by whose action on other structures a manifest change is produced. The similarity of these properties to those of some other agent might lead us to suspect the identity of their nature; but this would not give us any further information as to the abstract character of either. Our chief means of information, therefore, on the functions of the nervous system is derived from its effects on the other parts of the organism; and, by the attentive study of these, we may arrive at a knowledge of the conditions essential to its various operations, though we cannot precisely determine the nature of the operations themselves. Hence no enquiry into these functions can be complete which does not include an examination of the vital properties of the organs which it immediately influences: and this examination is particularly desirable at the present time, in order to correct the inaccurate notions which many have entertained regarding the dependence of organic life upon nervous influence; a doctrine which may be regarded as a remnant of the physiology of the Vitalists, which was scarcely less erroneous as a system than the chemical and mechanical theories which it displaced.

The property of *contractility* on the application of a stimulus is one of the most important of those enjoyed by living tissues. It is easily distinguished from that elasticity which is simply due to their molecular structure; the latter being retained as long as there is no evident decomposition, whilst the former is an essentially vital endowment. In the vegetable kingdom it is probably possessed, in a greater or less degree, by all the tissues actively concerned in the operations of nutrition and reproduction; and it is manifested under the influence of the normal stimuli of light, heat, air, moisture, &c., as well as, in some peculiar cases, in obedience to impressions of a mechanical nature. In the lowest and simplest animals, whatever degree of contractility is possessed appears to be almost equally diffused through the system; and we can neither discover in them any special structure peculiarly endowed with this property, nor anything resembling a nervous system fitted to call it into exercise. In proportion as we ascend in the scale, however, we find a distinct muscular structure evolved, in which the general contractility of the body becomes, as it were, concentrated; and, in proportion to its development and complexity, it supersedes the corresponding but more feeble powers of the remainder of the system. This view not only includes the animal function of locomotion, which in the highest classes depends entirely on the muscular system, (being accomplished in the lower by different means;) but also those parts of the functions of organic life which, in the more complex organisms, are subservient to it; and a very beautiful example of its application may be found in the circulation, which is evidently more dependent upon the impulse of its central muscular organ, and less aided by the vital properties of the general vascular system, in proportion as we ascend the scale.

The whole system of the vegetable is *immediately* dependent for its maintenance upon the action of external stimuli. All its vital properties are closely connected with the support of its organic life and the continuance of its race: all its energies are directed towards these ends. Each organ possesses a distinct vitality; since each, when separated from the rest, can perform its own function as long as the conditions essential to it are maintained. All are, however, blended and harmonized in the perfect plant by means of the circulating system; for the motion of fluid, unnecessary in the simplest organisms, where every part performs its share of all the changes necessary to the general existence, is required in those more complex structures which have a distinct organ appropriated to each function. There is in such beings no necessity for any other bond of union; nor do we find, in the ordinary phenomena of vegetable nutrition, any reason to suppose that such a one exists, since they can all be distinctly referred to the vital endowments of the several parts, brought into connexion with each other by the transmission of the nutritive fluid. But in some particular cases we observe evident motions performed in obedience, not merely to the ordinary vital stimuli, but to excitement of a mechanical kind. Such are the motions of the filaments of the Berberry, the closing of the fly-trap of the Dionæa, the flexion of the stalks of the pinnules and leaves of the Mimosa, and many others. In some of these we can trace a definite object connected with the performance of the organic functions, and it is beautiful to observe how this is attained. Nature, in effecting a new purpose, has accomplished it, not by adding an entirely new structure, but by modifying those already existing. In many of the instances to which allusion has been made, the sensible motions are the immediate result of the contraction of the irritated part; and we think it capable of proof that the property by which this is produced is but an exaltation of that common to most of the vegetable structure. In other cases, however, irritation of one surface produces motion of a distant part, as in the Dionæa and Mimosa; and this has afforded to the advocates of the existence of a nervous system in plants, a plausible ground for their arguments. But a full and cautious examination of the motions of the sensitive plant has shewn that this communication is effected through the medium of the circulating system, which not only serves as the bond of union between the organic functions, but even ministers to those which so nearly approach the endowments of animals. The contraction of the vesicles in the irritated part of the leaf forces a portion of their contained fluid through the vessels of the leafstalk into the intumescence at its base, by the distension of one side of which curvature is produced.* Other cases might probably be explained upon similar principles.

In the lower classes of the animal kingdom we regard the evident motions as scarcely less directly dependent upon external stimuli than those of plants, being, in fact, the result of the general diffusion of that exalted degree of irritability which is restricted in most plants to particular parts. We cannot believe the motions of the gemmules of the sponges to be much more voluntary than those of the sporules of some Algæ, which are equally active;† and the contractile tentacula of the Hydra and other Polypes would seem to act in obedience to direct mechanical stimuli. In proportion as the food of the animal is, like that of the plant, constantly within its reach, do we find the apparatus for obtaining it simplified and expanded through the system; but, when more laboured means become necessary for bringing it in contact with the organs of digestion, we find a special apparatus superadded to, not superseding, that already in existence. Thus, the Hydra may be regarded as *all stomach;* and the contraction of the tentacula by which its orifice is fringed, upon any object accidentally brought within their reach, would seem to us similar to that of the muscular parietes of the alimentary canal in higher animals, the functions of which are stimulated, and the continuance of those functions provided for, by the action of more complex systems directed to this especial end.‡

The question as to the degree of sensibility which is possessed by the lowest of the animal kingdom is one which we have not at present the means of solving. The motions which follow the impressions of external agents are our only means of judging of it; and the analogies which we have pointed out would seem to indicate that, if these motions are accompanied with sensation, they are not dependent upon it. In fact, it is as impossible to draw a distinct line between the animal and vegetable kingdoms, guided by the possession or absence of the powers of sensation and voluntary motion, as by any other criterion; since, when these powers are exercised in a very faint degree, their manifestations do not perceptibly differ from those of mere organic irritability.§ A nervous system would seem to be only required in a being possessed of a number

* See Dutrochet sur la Motilité des Végétaux, § ii.; and Mayo's Physiology, fourth Edition, pp. 10-13.

† For a very interesting account of these motions, see the memoir of L. C. Treviranus, in the Annales des Sciences Naturelles, tom. x.; and that of J. G. Agardh, in the same Journal, N. S. Botan. tom. vi.—Another corroboration of the view here taken is the fact that the ciliary motions by which these gemmules and many animalcules are entirely propelled, are known to be completely involuntary in the higher animals.

‡ We can scarcely express the pleasure we have felt in the perusal of Hunter's Croonian Lectures on Muscular Motion, published for the first time subsequently to the composition of the greatest part of the present article. In the first of these lectures we find the following passage, which will be seen to coincide entirely with the views expressed above: "These actions [of vegetables] are similar to what arise in many animals from external stimulus, more especially those not endowed with sensation, and also to the actions of many parts of animals which do not appear to be directed or stimulated by the brain and nerves; as the actions of a polypus, which has no brain, and the peristaltic motion of the intestines in the more perfect animals, which does not arise from the stimulus of the brain and nerves."—*Hunter's Works,* 1837, vol. iv. p. 201.

§ In a note to the passage just quoted from Hunter, Mr. Owen remarks, that observation of the motions of the living polype will shew that *all* its motions are not performed of distinct organs, whose actions are of such a character that they cannot be brought into mutual relation without a more immediate and effectual method of communication than the circulating system affords. "Mr. Hunter," says Sir G. Blane,* "by a happy turn of expression, calls the function of the nervous system *internuncial*. It is evident that some such principle must exist in the complicated system of the superior animals, in order to establish that connexion which constitutes each individual a WHOLE." Between the purely organic functions, the circulation, even in animals, is an important means of connexion; but the nature of the apparatus which is provided to ensure their continuance, and to adapt them to the conditions of animal existence, frequently (as in the respiratory organs) necessitates the intervention of the nervous system.

If we analyze, however, the connexions of the functions of organic life with those peculiar to the animal kingdom, we shall perceive that the actions of the latter may be divided into classes according to their more or less immediate relation with the former. Thus, in the very simplest cases in which muscular structure is introduced into the apparatus of organic life,—namely the heart and alimentary canal,—it is merely endowed with the property of vital contractility upon the direct application of a stimulus, and is not dependent upon nervous influence, though manifestly subjected to it for other purposes.† But were animals requiring food of such a nature as not to be immediately attainable, to be unfurnished with some other means of conveying it to their digestive organs than the organic contractility which the lowest and simplest possess, it is obvious that their existence could not be maintained: and, accordingly, we find the first appearance of distinct nervous cords in connexion with the buccal orifice of the alimentary canal. We can scarcely believe, however, that, as long as the whole surface is capable of receiving impressions, and no special organs of sensation have been evolved, this simple nervous system fulfils a much higher function than that which the spinal cord in vertebrated animals performs in relation to the actions of the constrictors of the pharynx and sphincter ani, the movements of respiration, &c. As we have before said, it is impossible to determine what degree of sensation and consciousness such beings possess; but we regard these psychical conditions as rather *superadded* to the chain of actions immediately connected with the organic funcin obedience to external stimuli, but that some have the appearance of being voluntary. Without wishing to deny this statement, (since to do so would reduce the being to the level of a plant,) we may remark that these animals probably act, like vegetables, in obedience to other stimuli than those purely mechanical; the application of which may escape the most careful observation.

* Medical Logic, p. 133.

† It will be seen that we employ throughout the Hallerian doctrine of the independent irritability of muscular fibre; not only because it is perfectly consistent with what we know of the vital endowments of other tissues, which the opposite doctrine is not, but because all the most recent and well-conducted experiments appear to confirm it. We may especially notice those of Dr. J. Reid, (Fourth Report of the British Association, p. 671,) who has shewn that the exhausted irritability of a muscle is recovered as speedily when its nerve has been divided as when it is entire; and those of Dr. Madden, communicated to the last meeting of the Association, who found that narcotics, acting through the nerves, destroyed their power of conveying stimulation to the muscles, long before the irritability of the muscle itself was impaired.

tions than as links necessary to their performance. In fact, we think that it will not be difficult to demonstrate that the class of associated movements whose object it is to supply their materials, (if we may use the expression,) and to get rid of their excrementitious products, not only are independent of the will, though in part under its control, but do not necessarily involve sensation as a condition of their performance. The movements of respiration, for example, are not, strictly speaking, a part of that function, any more than is the action of the heart: the former ensure a constant supply of pure air, and the removal of that which has been vitiated; whilst the latter maintains a flow of venous blood to the lungs, and withdraws that which has been arterialized. If either of these conditions be impeded or checked, the real function of respiration is proportionally impaired.

In proportion to the exaltation of the sensibility, and especially to the evolution of the organs of special sensation from those more general forms which are presented in the lower parts of the animal scale,* do we find that the supply of materials for the maintenance of the organic functions is made to depend remotely upon their exercise; but still the actions immediately connected with the sensations excited by external conditions would appear to have but little of a voluntary character, and to involve no intellectual process whatever. These actions are usually denominated *instinctive*, and have been distinguished from the *automatic* motions, not merely of the heart and alimentary canal, but of the muscles of respiration, defecation, &c. We confess, however, that we do not see why the term *instinctive* should not be applied to *all* the actions which are performed in *direct respondence to an external stimulus;* and, in fact, we shall hereafter render it extremely probable that some of those which have been regarded as excellent examples of the restricted class to which we have just alluded,—as, for instance, the act of sucking in the infant,—are really not only involuntary, but not dependent on sensation.†

The highest class of muscular motions excited by the nervous system is that which is strictly voluntary, being produced by operations of the intellect, which are only remotely dependent on sensation. The will is capable, if sufficiently powerful, of modifying or controlling the involuntary actions to which we have already alluded; and every one must be aware of its influence in morbid states of the system. Thus, the muscular movements in hysteria and tetanus are, in their causes, equally independent of the will: in the former disease, however, a predominance of the will over the power which stimulates them may prevent their performance, although a deficiency in the exercise of the will too frequently

* There appears little doubt that a diffused sensibility to light and sound exists in animals which present no special organs of vision or hearing.

† It has been objected, by a distinguished physiologist, to this use ef the term *instinct*, that "it is employed, both in common and scientific language, to denote a *mental* act; something of which our own minds are conscious, or of which we believe that other minds (whether of man or animals) are conscious." To this we reply, that the term instinct belongs to a confessedly doubtful and debateable ground; that any writer is therefore at liberty to extend or limit it as he pleases, provided he defines his own meaning; that *we* (as will subsequently appear) do not regard as *dependent* upon sensation, or any other act of mind, many actions which are usually considered in this light; and that many writers speak of instinct as a "blind impulse," an "organic necessity," &c., phrases which certainly do not imply any "mental act."

allows them free play; whilst, in the latter, the strength of the stimulus is such that no effort of will can resist or overcome it. The actions which are the result of intellectual processes appear, in general, but remotely connected with the gratification of the bodily appetites, when compared with those which are purely instinctive; and, in proportion to the difficulties which attend the acquirement of the means of maintaining the organic life, do we find the mental powers, adapted to a variety of conditions, predominate over the instincts whose scope is limited to a few.

The nervous apparatus being composed of two distinct structures, it is a matter of some importance to ascertain their respective functions before entering upon the discussion of the offices of particular portions of the system; and accordingly we find the second chapter of Mr. Grainger's book devoted to an examination of the "Properties of the Grey and Fibrous Substances." The opinion entertained by Mr. Grainger is, that the grey matter is the sole source of power, and that the white fibres are conductors only. In support of it he adduces the great vascularity of the former as compared with the latter, and the well-known immediate dependence of the functions of the nervous system upon the continuance of the circulation. This we regard as a very fair argument, and it is one which has been much insisted on from the time that the injections of Ruysch first substantiated the extraordinary vascularity of the cortical substance: it by no means proves, however, that the cortical substance is the *exclusive* originator of power; still less that it is the peculiar seat of sensibility.* Mr. G. also urges, in confirmation of his opinion, that "the grey matter increases in quantity in the exact ratio of the nervous energy." This we believe to be a general fact, but by no means so universal as Mr. G. supposes. We find that in those of the lower Articulata, whose motor organs appertain equally to each segment, the ganglia are nearly uniform in size throughout the body, as in the Myriapodes and larva condition of the insect tribes. In the perfect insect, where the legs, which in the caterpillar belonged to the nine posterior segments, have altogether disappeared, and where, in addition to the legs of the thoracic segments, two pairs of wings have been developed, the ganglia are found to be more or less concentrated in the anterior region, as Herold long ago demonstrated : but these enlargements are not directly connected with the motor nerves, or at least with the column believed to have that office, which passes over the ganglia without entering them. Mr. Newport has recently shewn, in a paper read to the Entomological Society, that there are similar enlargements on the motor columns of one of the Tenthredos, or saw-flies, where they pass over the ganglia, and where large nerves are given off; and stated that these are

* We can scarcely regard that as a valid argument against this hypothesis which we have known urged by more than one eminent physiologist,—that the possibility of exciting motions by stimulating a nerve separated from the brain disproves the idea of the origination of the motive power by the grey matter. A similar argument would lead to the inference that *no part* of the brain or spinal cord is the source of that influence by which muscular motions are excited; and that the impressions which produce *sensations* are not transmitted from the peripheral extremities of the sensory nerves, because similar sensations may be produced by pinching a nervous trunk disconnected from them. These effects are produced by the artificial stimulus *imitating* the changes ordinarily produced by the natural one.

entirely composed of ***fibrous*** matter, no grey matter entering into the structure of these columns. It is impossible to come to a definite conclusion on such contradictory testimony, and with such imperfect evidence.

That the office of the white matter of the nervous centres is similar to that of the nervous cords themselves, it would seem fair to infer, from their similarity of structure, were it not that, on subjects of this kind, we are constantly obliged to feel our uncertainty in arguing as to similarity of function, from the insufficient data which our imperfect means of research afford. That the *white matter*, wherever it exists, has a tubular structure seems now generally acknowledged; but whether the tubes are essentially different in character in the substance of the brain and that of the nerves must still be regarded as a controverted question. The *cortical* substance of the brain is stated by Ehrenberg to consist of a minutely reticulated plexus of blood-vessels, in the interstices of which are seen a number of free granules, apparently similar to the small colourless globules of the blood: these resemble in size, though not always in form, the nuclei of the red particles, and are believed by Ehrenberg, as well as by Müller, to be identical with the globules of chyle and lymph. In the cortical substance, especially towards its junction with the medullary, a delicate fibrous appearance is seen, which is apparently the commencement of the tubular structure of the latter; and it is conjectured by Ehrenberg that the detached granules may pass into the open mouths of these tubes. He has remarked also that the red globules in the vessels of this part are smaller than usual, and are partially deprived of their external envelope. In watching the early development, also, of the cerebral substance, he found it to consist of a coarse granular matter, from which cylindrical tubes were produced at a later period.

Highly appreciating, as we do, the importance of an accurate and extensive series of such observations, we cannot yet think them sufficient, either in number or uniformity of tendency, to bear the weight of any important inferences; and we think that many more facts must be collected from the sources laid open by anatomical research and pathological observation, before we can regard that as *proved* which, at present, is, certainly, at most but a plausible theory. When speaking of the structure of the spinal cord in Vertebrata, we shall adduce one or more facts apparently adverse to it; but we may recommend to the attention of our readers Mr. Grainger's own statement of his argument, with which we have no fault to find but its narrow basis and its onesidedness.

We shall now briefly sketch the principal varieties which are met with in tracing the arrangement and combination of these elements in the different divisions of the animal scale.—The group of Acrita is regarded as comprehending those classes in which no definite nervous system can be discovered. It is generally believed that, in the animals which belong to it, the nervous matter is present in a "diffused form,"—that is to say, incorporated with the tissues; but we see no reason whatever for such a gratuitous supposition. The simplest office of a nervous system is to establish a communication between parts specially modified to receive impressions, and others particularly adapted to respond to them. Where every portion of the body has similar endowments, there can be no object in such a communication; just as, where every part of the surface is equally capable of absorption, and every part of the tissue equally permeated by nutrient fluid, there is no necessity for a circulating system. Many beings comprehended in this group manifest much less irritability than that possessed by the Mimosa or Dionæa; and the mere possession of this property to any amount may be quite independent of a nervous system; as was strongly and, we think, satisfactorily argued by Hunter, in the Croonian Lecture already referred to. The life of the Sponge and the Polype we regard as much more organic than animal; and such beings form the natural links of transition from one kingdom to the other.

The earliest distinct trace of a nervous system consists of a simple cord without ganglia, which in the Radiata is circular, in the Articulata longitudinal. As we rise in the scale, however, we find grey matter in the ganglia, which, in the higher Radiata, are disposed at definite intervals round the circle; and, in the Articulata, generally correspond with the division of the body into segments. In proportion as the endowments of all the segments are alike, the distribution of the ganglia is uniform; but, as the organs of special sensation increase in number and perfection, a concentration of the ganglia towards the head is evident; and, according as the locomotive apparatus of any particular segment is developed to a greater extent than the rest, does the ganglion of that segment exhibit an increased size. Anatomical research has shewn that the knotted nervous cord of the Articulata is not simple, but consists of distinct tracts; and that, independently of this, there is a system especially distributed to the respiratory apparatus, taking its origin from the cerebral ganglion, and another peculiarly connected with the viscera. It is usually believed that, of the tracts of the ganglionic cord, one is for sensation and the other for motion; the sensory nerves alone entering into the ganglia, and the motor column passing over them. If the view which is taken by Mr. Grainger of the structure and functions of the different parts of the spinal cord should hereafter be substantiated, we must seek for a different explanation of the endowments of the divisions of the nervous column of the Articulata: we shall presently allude to one that appears to us more consistent than that just given. In those Articulata whose several divisions have equal nervous endowments, the separation of the segments appears to produce little immediate influence upon their locomotive powers; and the movements effected by each portion are so simple and uniform that the general will would have little else to do than to control and direct them. In the articulated classes we see the instinctive propensities developed to their full extent, and the animal appears to be almost entirely guided by them. The perfection of the locomotive apparatus appears to be the governing principle in the structure of this division of the animal kingdom; and, accordingly, we find not only the nervous system but almost every other part of the body arranged on a symmetrical design.

In the Mollusca, on the other hand, the nervous system is connected with the maintenance of the organic life rather through the medium of the organs of sensation than those of locomotion. The extraordinary development of the digestive system of these inactive and often fixed creatures required that the animal functions should be immediately connected with the entrance to the alimentary canal. Accordingly we find that the ganglia are disposed around the œsophagus and connected with others scattered through the body; and, in proportion to the development of the organs of special sensation do we find the nervous centres brought together into a situation superior to the œsophagus, so that in the highest cephalopodes the distribution of the elements differs little from that which is found in the lowest of the vertebrata.

In the Vertebrated classes we find united the forms which the nervous system has presented in the articulata and mollusca. The spinal cord, composed of cortical and fibrous matter, like the chain of ganglia in the former, traverses the body longitudinally, ministering particularly to the function of locomotion; whilst the ganglia connected with the organs of the senses, on which the supply of nutriment immediately depends, are placed in the neighbourhood of the alimentary canal,—not however, surrounding the œsophagus as in the mollusca, but entirely above it. In fishes the hemispheric ganglia and cerebellum make their first appearance as distinct organs; but they are quite subordinate to the cerebro-spinal axis and its ganglia. It is interesting to remark that the different forms which the nervous system assumes in this class, distinctly correspond with those which are typical of the sub-kingdoms whose peculiar characters are combined in it. Thus in the eel and other vermiform fishes, the spinal cord is of nearly uniform size throughout, and the cerebral ganglia are scarcely more prominent than those of the leech or caterpillar. In proportion as distinct organs of locomotion are developed, do we find enlargements of the spinal cord corresponding with the origins of their nerves, just as in the ganglionic column of insects; and where the anterior members are very powerful, as in the *Trigla* (gurnard), the spinal cord presents enlargements evidently ganglionic.* In such fishes as the *Lophius* (frog-fish), on the other hand, whose head and nutritive organs are enormously developed at the expense of the rest, and the locomotive apparatus principally in the anterior region, the nervous centres seem entirely confined to this division of the body; for the true spinal cord quickly separates into a bundle of distinct nerves analogous to a cauda equina.

We shall not minutely trace the progressive complications of the nervous system through the different classes of vertebrata, since our readers have other means of becoming acquainted with them. In proportion as we ascend towards the higher mammalia do we usually observe an increased development of the cerebral lobes or hemispheric ganglia with respect to the cerebro-spinal axis and the ganglia immediately connected with the nerves of sensation; the surface becomes convoluted, so as to augment the quantity of cortical substance; and the complexity in the arrangement of the fibres of the medullary portion greatly increases.

The anatomy of the spinal cord of vertebrata has been an object of frequent research; and the views taken of the relations of its different parts have necessarily varied much with the physiological doctrines which have been appended to them. According to the latest description of Sir C. Bell, the anterior roots of the spinal nerves are connected with the anterior columns upon which he believes motion to be dependent; whilst the posterior roots arise, not from the posterior columns, as he originally

* Mr. Owen informs us that these ganglia are found, in relation to sensitive organs, superadded to the pectoral fins. They would thus seem to resemble the thoracic ganglia of insects; and, if the latter should be shewn to be excito-motor, we suspect that these would prove to have a similar function. (See p. 500.)

supposed, but from the lateral. Other anatomists, however, take a different view; and Mr. Grainger (*Observations*, p. 30,) agrees with Mr. Swan in the belief that the anterior and posterior lateral fissures appear definitely to limit the two roots; or, in other words, that the nerves are entirely connected with the lateral column alone. Our own observations do not accord exactly with either of these views: but we are far from asserting that we are certainly right, and these eminent anatomists certainly wrong; as we have always regarded the determination of the precise mode in which the roots of the spinal nerves are connected with the nervous matter of the cord as one of the most difficult problems in anatomy.

The following is Mr. Grainger's account of the alleged connexion of the roots of the nerves with the grey matter of the cord.

"After the two roots have perforated the theca vertebralis, and so reached the surface of the cord, it is well known that their fibres begin to separate from each other: of these fibres some are lost in the white substance, whilst others, entering more deeply into the lateral furrows, are found to continue their course nearly in a right angle with the spinal cord itself as far as the grey substance in which they are lost: but this arrangement has no resemblance to the distinct division into fasciculi depicted by Mr. Mayo; on the contrary, it is with great care only that small delicate and individual threads or striæ, as it were, are traced dipping into the lateral fissure, and at length joining the grey matter." (*Grainger*, p. 34.)

"It is extremely difficult, owing to the delicacy of the parts, to determine the exact relations which exist between the above filaments and the grey matter, but in a few dissections I have been able to perceive these fibrils running like delicate striæ in the grey substance. In one instance, the fibres being more distinct than usual, an appearance was presented having a remarkable resemblance to that which is seen on making a section of the corpus striatum in a recent brain after the method of Spurzheim. My friend and colleague, Mr. Cooper, in this case counted distinctly five separate fibrils passing from the anterior root of one nerve, and there were some other fibres derived from the same root which were not so plainly seen." (P. 35.)

"From careful dissection, I am convinced that it is only a part of the fibres belonging to the two roots which are attached to the grey substance, and that a considerable number of threads are lost in the fibrous part of the cord. The exact mode of their connexion, however, with this latter substance is not known." (P. 37.)

Without pronouncing on the accuracy of this description, we do not think that Mr. Grainger was aware when he stated his results how nearly they corresponded with those of Bellingeri, published many years ago.* He quotes this author as having "attributed both to the anterior and posterior roots a triple origin; the former arising by two of its roots from the white fibrous parts, and by the third root, *perhaps*, from the grey matter; the latter arising by two roots from the fibrous part, and by the third from the posterior horn of the grey substance." Now the fact is that this description applies to the spinal cord in the human subject, in which Bellingeri confesses the difficulty of tracing the anterior root into the grey substance, although he maintains that several of the filaments pass into the antero-lateral grooves, where the anterior cornu of the grey matter approaches the surface; perhaps from the cause mentioned by Mr. Grainger, that the lapse of a few hours after death softens the parts so as to render this minute dissection impossible. But it is distinctly stated by Bellingeri,† that he *was able* to trace the connexion of the fila-

* De Medullâ Spinali. Aug. Taurinor. 1823. † Op. cit., p. 160.

ments of the *anterior* root with the *grey matter* at the inferior part of the lumbar and superior part of the sacral region of the spinal marrow in the ox. Mr. G. does not tell us that he has traced this connexion in the human subject; and as the plates which he has given are taken from the dog, we infer that he has not. We do not see, therefore, that he can be said to have made any particular advance in the state of our knowledge on this subject: his researches possess merit, as tending to confirm or correct certain previous statements; and the mode in which his views are brought forward will probably excite more general attention than was given to the statements of Bellingeri.

We will now briefly state the result of our own observations and dissections; only remarking that the observations relative to the connexion of the roots of the nerves with the grey matter of the cord are drawn from recent examination of the spinal cord in the dog, made within a couple of hours after the animal's death, according to the suggestions of Mr. Grainger, and with the other precautions recommended by him.

In regard to the *posterior roots,* on opening the postero-lateral fissure at several places along its length, we could perceive, opposite the attachments of some of the pairs of nerves, though not of all, white streaks running into the grey matter. These striæ were decidedly nothing else than white nervous matter, (certainly not blood-vessels:) they were exceedingly delicate, but distinguishable by colour from the grey matter into which they penetrated; they ran from the lips of the fissure, and nearly at right angles to them into the grey matter. It is not improbable that these white tracts were continuous with some of the funiculi of the nervous roots; but this point we could not determine. In their aspect they correspond more with Mr. Grainger's description than with his figures, to the broad stripes represented in which these exceedingly fine striæ have no resemblance. In regard to the other and principal attachment of the posterior root,—viz. to the white substance,—our observations, as already stated, lead us to differ from Mr. Grainger's views, which are those of Sir Charles Bell. These anatomists restrict this attachment to what is usually called the lateral column of the cord; or, in other words, to the anterior border of the fissure. We are satisfied, however, that this description will not apply to all the nerves; for, in regard to some of the pairs, (for instance, those going to the upper extremity, which have very large roots,) the bundles of the posterior root have most certainly an attachment also to the posterior column.

As to the *anterior roots,* we have not yet been able to trace them beyond the surface of the cord; but we do not, therefore, dispute the accuracy of the descriptions of Bellingeri and Mr. Grainger. We must also here say that we have never yet been able to satisfy ourselves as to the existence of a fissure at this part of the cord. But, admitting that the irregular furrow on the surface, where the anterior roots come off, and the peak of grey matter corresponding to it within, really indicate a division of the cord at this part into an anterior and lateral column, we suspect Mr. G. is in error when he limits the attachment of the anterior roots to the so-called lateral column and the grey matter. As to the alleged connexion of these roots with the grey matter, we do not mean to deny it, although we have been altogether unable to perceive it; but the scattered mode of origin of the funiculi composing the anterior roots,—some being placed more anteriorly, and others further back,—seems to us to be much opposed to the view that the anterior roots, in their attachment to the white matter of the cord, are confined to the lateral columns.

Mr. Grainger is of opinion that all those fibres of a spinal nerve which are not lost in the grey matter ascend, in an uninterrupted and independent course, to the brain, and exclusively form, as he supposes, the internunciate chords of sensation and voluntary impulse.

Now, though it would be difficult to demonstrate the incorrectness of this view by dissection in the human subject, yet it is evidently open to, if not subverted by, the following objection,—a sufficiently obvious one, but not foreseen, it would appear, by Mr. Grainger. Say that the spinal cord has a certain thickness where it communicates with the 28th spinal nerve, it must be thicker where the ascending fibrils of this nerve are added to those of the 27th; and, though at first the enlargement would be imperceptible, yet the increase of size should be obvious at the part where the cord contains the accumulated fibrils of all the spinal nerves. In rebutting this argument, an opponent might take advantage of the partial enlargements, due, according to Grainger's theory, to nervous matter in association with the automatic or reflex fibrils, and thus dispose of this additional matter: such an objection is not, it must be admitted, worth much. But, let us take a boa-constrictor, where the spinal cord has no partial enlargements; the cord is not thicker where it joins the brain than at the posterior quarter-part of the body: this fact seems to be incompatible with Mr. Grainger's ideas of its structure.

The following is the account which Mr. Grainger gives of the cerebral nerves:

"By minute dissection, it becomes apparent that these nerves resemble those of the vertebral canal, as from their similarity in function might be anticipated, in being attached both to the grey and fibrous substances. Their connexions, indeed, are in some respects more easily traced, in consequence of several of them arising in large trunks, whilst the two roots of the spinal nerves are spread out into single threads when they reach the cord. In this respect the third or motor oculi is particularly distinguished, and, as it may with strict propriety be received as the type of the motor nerves of the spinal cord, its anatomical relations demand special consideration. It is stated by Mr. Mayo that this nerve arises by many fibrils from the black matter in the crus cerebri. This account is perfectly correct as far as it extends, but it is not complete; for, as Mr. Solly has shewn, some of the fibres are attached to the motor cord in the pons varolii. But what is particularly interesting, is that after the fibres have spread out into the grey matter or locus niger, some of them may with care be followed into the fibres which constitute the upper portion of the crus. Now this part of the crus cerebri has been described by Sir C. Bell as receiving certain fasciculi derived from the posterior or sentient column of the spinal cord where it forms the calamus scriptorius of the fourth ventricle, and which subsequently pass upwards through the thalami nervorum opticorum to the cerebral hemispheres." (Pp. 38-39.)

As the optic nerve is connected in its origin with this tract, a relation is established between it and the motor oculi, which is considered as affording a satisfactory explanation of the facts established by Mr. Mayo regarding their mutual action in producing closure of the pupil. Mr. G. thinks that by a similar continuity of fibres between the posterior and anterior roots of the spinal nerves, and of the portio major and minor of the fifth cerebral, established through the grey matter of the cord, may be explained the mode in which impressions made on the nerves of the skin are transmitted to those of the muscles; this continuity he has not succeeded in tracing, but only infers it from analogy. The close approximation of the origins of the portio major of the fifth and of the portio dura of the seventh is another similar fact of great physiological interest; and was stated by Mr. Mayo some years ago, with several others of the same character, as leading to the belief that "nerves of motion take their rise from the same region or segment with those sentient nerves which transmit the impressions by which their action is usually regulated." We shall presently see that Mr. M.'s explanation will not apply to all the phenomena of sympathetic actions; although we have little doubt of the fact that a direct relation effected by a continuity of structure exists between all the excitor nerves and the motor nerves respectively called into action by them.

Mr. Grainger compares the constant relation between the size of the spinal cord and the nerves proceeding from it in the different tribes of vertebrata, with the variable proportion which the cerebral hemispheres present; and hence draws a fair argument for the independent power of the former. The enlargement of the column at the origins of the nerves of the extremities seems also to lead to the same inference. The doctrine founded by Mr. Grainger upon these and other anatomical facts will be best stated in his own words. He regards the spinal cord as composed of two structures anatomically and physiologically distinct from one another.

"One of these structures consists of the grey matter, confined, it must be recollected, not merely to the vertebral portion of the cord, but extending into the cranium, as far as the striated bodies, the optic thalami, and the optic tubercles. Now it has been shewn [?] that the grey substance, in general, is the source of the nervous power; and I believe it is further susceptible of proof that the portion of that substance which is lodged in the spinal cord is the source of the peculiar powers possessed by that organ; that, in fact, it constitutes, with the nervous fibres attached to it, the true spinal cord, the existence of which, as a structure independent of the brain, was first declared by Dr. M. Hall. The second portion of the spinal cord consists of the white fibres, all of which, after a most complicated disposition, seem to extend to the convolutions of the cerebrum and the layers of the cerebellum; it may, therefore, with propriety be called the cerebral part of the cord." (Pp. 45-6.)

Connecting this view with his previous statements he infers that

"In the compound nerves of the body there are in reality four instead of two different classes of fibres; and when the physiology of these parts is considered, it will be made apparent that these several fibres transmit different impressions: that of those going to the brain, the fibres derived from the sentient nerves transmit impressions which excite sensation, and those belonging to the motor nerves, volition; that of the fibres attached to the grey matter of the cord, those derived from the sentient nerves transmit impressions made on the skin to the true spinal cord, the peculiar power of which they excite; whilst those derived from the motor nerves transmit to the muscles the effects of the power thus excited." (Pp. 46-7.)

The connexion of the cerebral nerves he regards as not dissimilar.

"It is well known that whilst such nerves are admitted by some physiologists, the majority of writers in the present day contend that all the nerves contained within the cranium are spinal nerves. Neither of these opinions is correct; for each of the cranial nerves is in reality composed, like those attached to the vertebral part of the spinal cord, of a true spinal and a true cerebral portion: the former being attached to the prolongation of the grey matter of the cord, and the latter to the sensiferous and volition fibres which ascend to the grey matter of the cerebral convolutions. The only nerve which perhaps consists of cerebral fibrils alone is the olfactory." (Pp. 47-48.)

It is well known that Dr. M. Hall proposes to admit as a part of his system of nervous physiology a set of excito-motor fibres distinct from those connected with sensation and volition; this admission we strongly opposed when formerly treating of this subject, and we cannot see that the facts brought forward by Mr. Grainger are yet sufficient to warrant it. It must be recollected that the inference which he deduces from them is entirely dependent on the opinion maintained regarding the respective functions of the grey and white nervous matter; and though we may consider the preponderance of evidence to be rather in favour of the doctrine held by him, we cannot consider this doctrine as resting on proofs sufficiently valid and stable to allow us to employ it as the foundation for another superstructure. Its application to comparative anatomy is not without its difficulties; thus Desmoulins contends that the grey matter does not exist in the spinal cord of fishes and reptiles; but that there is in the centre a canal filled with a serous fluid. "This statement," says Mr. G. "is, I believe, altogether erroneous; for, although it may be impossible to demonstrate in the cord of these animals the presence of a substance distinguished by its grey colour or consistence,* this circumstance is not sufficient to prove that a structure possessing the same properties as the grey matter does not exist." This explanation, we presume, would be applied by Mr. Grainger to the fact which we state on the authority of Mr. Newport, that in insects nothing but fibrous matter exists in those portions of the cords which are believed to be the motor, and he thinks not in the sensitive portions, except at certain points where they surround and inclose nodules of grey matter to form the ganglia;† but we cannot regard Mr. Grainger's explanation as altogether satisfactory; since the vascularity of the grey matter is not only its evident characteristic, but the source from which its peculiar properties, whatever they are, may reasonably be regarded as derived. Some of the difficulties arising from the examination of the nervous system of insects may perhaps be explained by an hypothesis of Dr. Hall's, which has a plausible aspect, when taken in connexion with Mr. Grainger's. He regards the ganglionic column of insects as strictly *excito-motor;* and the tract which does not enter into the ganglia, and is usually regarded as *motor*, he seems to consider as conducting the motive influence of volition.‡ If

* We have the authority of Mr. Owen for stating that grey matter does exist in the spinal cord of the turtle, crocodile, and python: and the same is stated by Mr. Swan, in the recent part of his "Illustrations of the Comparative Anatomy of the Nervous System."—REV.

† The manner in which the sensitive portions of the cords inclose the nodules of grey matter to form the ganglia, is shewn in the plates belonging to Mr. Newport's Paper in the Philosophical Transactions, 1834, part ii., plates 15 and 16, figures 32, 33, and 36. The nodule of grey matter is there shewn to be distinct from the sensitive portions of the cords which surround it.

‡ Since the above was in type, we have observed, in Dr. Hall's "Lectures on the Practice of Medicine," at present in course of publication, the following experiments bearing on this subject:—"I took a lobster, and laid bare the nervous columns. I first stimulated one of the ganglionic nerves: the muscles to which it was distributed, and *they alone*, were contracted. I then stimulated a ganglionic nerve. Muscles, both *anterior* and *posterior* to the part stimulated, were excited into combined action." (*Lancet*, Feb. 3, p. 650.) A great number and variety of experiments will, we think, be necesconjecture have any truth, we should regard this tract as corresponding with the cerebral portion of the spinal cord supposed by Mr. Grainger to exist in vertebrata; and therefore as ministering both to sensation and motion. There is certainly much correspondence between the structure of the double cord in the Articulata in general and the medulla spinalis of the Vertebrata, inasmuch as the ganglia always contain cineritious matter; whilst the cords which connect them and the nerves which proceed from them are almost entirely transparent and colourless, and differ very much in appearance from the granulated substance of the ganglia. Mr. Mayo may reasonably lay claim to having some years ago arrived at an opinion similar in its general bearings, which we quote from the second edition of his Physiology, published in 1829. "The cords which unite the nodules in the nervous systems of invertebral animals, we may presume, are intended to transmit reciprocally the influence of the different segments of the nervous system from one to another. The white fibrous strings which form the outside of the spinal cord in man and vertebral animals, have probably the same office." This opinion harmonizes well with the fact, not of very unfrequent occurrence, that when the arms have been palsied or permanently contracted without the lower part of the body being affected, the segments connected with the nerves of the former have been found upon dissection wasted, whilst cords of white matter have passed over them to the lower part of the spinal cord which has exhibited a natural appearance. On the other hand, it may fairly be argued that the enlargement of the cord where the nerves of the extremities are given off is produced by the accumulation of grey matter; and as these nerves are seldom put in action except voluntarily, there would seem but little cause for strengthening at these parts the supposed excito-motor portion of the cord. Again, there is no particular accumulation of grey matter in connexion with the origins of the respiratory nerves, whose associated movements, depending on the spinal cord alone, are in constant operation. Although we do not regard these and other similar facts as disproving Mr. Grainger's theory, we consider ourselves justified by them in withholding for the present our assent to it.

We believe that we have now put our readers in possession of all the essential facts which recent anatomical researches have contributed towards the elucidation of the different physiological questions on which we must now enter.

Before going further, however, we must specify the use that we intend making of certain terms, which, from their conveying ideas of an abstract rather than a tangible kind, have been peculiarly liable to be understood in a variety of senses. In fact, the difficulty of defining these terms so as to give a precise idea of the functions of the nervous system has been a fruitful source of unprofitable discussion, and has much retarded the progress of real knowledge. We cannot describe the properties of a sensation as we do those of the bile or the urine; and yet the production of the former is as much a function of the nervous system as the secretion of sary to the determination of the precise functions of these nerves; and we cannot regard these as either decidedly confirming Dr. Hall's hypothesis, or as opposing the improvement of it which is above suggested.—Rev.

the latter is of their respective glands.* The chemist judges of the properties of secretions by observing their action with various reagents whose characters are known; whilst the psychical changes produced by sensation are more obscure than the very object of our examination. The term *sensation* is used by metaphysicians in two distinct senses; first, to denote the communication to the conscious mind of an external impression conveyed to the sensorium; and second, to designate the mental condition which is the immediate result of such communication. In other words, it may imply either the *act*, or the resultant *product*. The term *secretion* is in exactly the same predicament. It is evident that *consciousness* is necessary to the production of any change in the state of the mind; and, therefore, to speak of sensation without consciousness is a contradiction. At the same time consciousness is not identical with sensation as some writers have maintained, but is that condition of the mind which renders it open to the reception of sensation; just as, to carry out the parallel we have already employed, the peculiar vital properties of an individual gland render it prepared to secrete a particular product when the blood which contains the elements of that secretion circulates through it. The term sensation is often confounded by physiological writers with *sensibility*, which is the capability of receiving impressions so as to give rise to sensation. We often find the brain or spinal cord spoken of as the seat of *sensation*, when the *property* of sensibility is all that can be strictly allowed to exist there. On the other hand, we find Mr. Grainger saying (p. 119,) "There is but one kind of *sensibility* possessed by animals; that, namely, which is *perceived* by the mind." The *property* of sensibility cannot become an object of perception until it manifests itself by an act of sensation, the *result* of which gives rise to the mental condition termed perception; the correct expression would therefore be, "that which is indicated by sensations of which the mind is conscious." The term *motion* has been used with similar inaccuracy; for we find it used by physiologists in three distinct senses; namely, the *capability* of producing motion (a property inherent in certain parts of the nervous system), the *act* of producing motion, and motion itself or the *result* of the act. We have entered into these details simply with the view of shewing the prevalent errors on this subject, and of enforcing the necessity of attention to accuracy among all those who aim at rendering metaphysical physiology as definite as the more tangible parts of the science.

Just as it is the function of the absorbents to convey to the centre of the circulation from all parts of the surface or interior of the body the fluid which they have imbibed, is it the property of certain of the nervous fibrils to transmit to the central sensorium the changes produced at their extremities. Until the mind takes cognizance of these impressions no sensation is produced; to whatever motor changes they may give rise, as long as the mind is unconcerned in them, their character is the same; but the consciousness of an impression is an act of *sensation;* and here the purely mental functions commence. Few physiological writers keep in view the distinction which is metaphysically required between sensation and per-

* We wish to be understood as referring to no distinctly *mental* act, but merely to that communication to the mind of an external impression which most physiologists believe to be effected by an organic change in the nervous matter.

ception, and the terms are frequently used synonymously. This is, however, a great error, since *perception* is, strictly speaking, the act of forming that elementary notion of the properties of the body occasioning the sensation, which is quite different from the sensation itself, and which must intervene before any reasoning process can be founded upon it.*

All acts of thought are either immediately or remotely dependent upon sensations; and if all its inlets were closed, the mind would remain dormant like the seed buried deep in the earth. The activity of the mind is just as much the consequence of external impressions by which its faculties are called into play, as is the life of the body the result of the excitement of its several vital properties by external stimuli; and just as many animals are capable of retaining a certain degree not only of vitality but of vital action, when deprived for a time of these stimuli, (as in hybernation,) so could the mind which had once been roused retain its powers by the recall of its former sensations, though debarred from the excitement of new ones.

We shall presently endeavour to shew that a certain class of muscular actions is as closely and necessarily connected with the excitement of *sensations* (these being usually of a *special* kind,) as another is with the stimulus of *impressions* only; and we think that it betokens a hasty and partial view to assert, as Mr. Grainger does, that, "if sensation be at all operative, it can only be so by exciting the will; for to say that an involuntary muscular action is the result of perception is to deny the involuntary character altogether." (P. 84.) All physiologists allow that the directly *emotional* actions, which are of course dependent upon changes excited by sensation, are independent of the will, and frequently opposed to it. It is only when these emotions are strongly excited, however, that the actions performed in obedience to them have this character; if less vehement, or partially subdued by the will, the excited emotion merely stimulates the intellectual processes to the formation of a desire (of which it then becomes an element) from which an act of volition results. The distinctness in the channels of emotional and volitional actions is beautifully evinced by pathological cases; since experiment is here entirely unavailing. We have, in our last volume (p. 500), recorded an instance in which the control of the will over the muscles of the face was entirely lost; yet, when a mental emotion was excited, the movements of smiling and laughing were performed as in the natural condition. Dr. M. Hall also mentions (*Memoirs*, p. 102,) a case of hemiplegia, in which the paralysed hand and arm were contracted and convulsed in the most extraordinary manner whenever the patient was excited by meeting an acquaintance, or in any similar way.

We shall not enter into a metaphysical argument on the nature and sources of *volition*, but leave it to the common sense of our readers to inform them how far this mental condition, resulting from the exercise of the intellectual faculties and the stimulus of the moderately excited emotions, is under the control of the individual himself: but we must observe, that in man its power may be exercised not only upon the body but over the mind. In the latter it maintains a general regulation over

* The term *perception* has, like *sensation*, a double import; being used to designate the *act* of forming the notion, and the *resultant notion* itself.

the passions and emotions, recalls and combines ideas into conceptions, and controls the sequences of thought. On the former it can only act by producing that change in the nervous system, which, when propagated to the extremities of the motor nerves, excites muscular contraction; and the result of this contraction is either evident motion or the resistance and prevention of the involuntary movements which would otherwise occur. This change, effected in the body by the influence of the mind, and transmitted from the centre to the circumference, is manifestly reciprocal to sensation: it has been designated as the influence of volition, but this is an objectionable term, since (as we have just observed) volition may be exercised in the direction of the mind as well as of the body. The term *motive action*, which has also been suggested, would be preferable, not only on this account, but as applicable to the similar change in the nervous system excited by purely emotional acts of mind. It is, therefore, extremely incorrect to speak either of the brain, the spinal cord, or the nerves, as the seat of motion: none of them can be regarded as such, any more than the retina or the optic nerve can be said to be the seat of sensation. It is the function of these parts to excite and propagate those changes which, by their action on other parts, produce the effects in question.

The emotions, passions, and propensities are all conditions of an ana logous nature, but vary as to the degree in which they are connected with the operations of the intellect, or with the performance of the organic functions. The acts *immediately* resulting from all of them, however, are carefully distinguished from those of volition; though, when only moderately excited, they act by supplying motives to the will. In proportion to the degree in which they predominate, whether in health or disease, does the being lose his freedom of will, and his actions approach towards an instinctive character.

It will readily be perceived how great is the tendency to union in these two sources of action, volition and emotion. A purely instinctive action is altogether involuntary; whilst a purely voluntary action is the result of intellectual processes alone, and derives no stimulus from the propensities or emotions. A large proportion of the actions of man are of a mixed character, and result from the influence of the natural passions on the intellectual operations. It is in these alone, therefore, that the propensities act through the will; since the observation of the habits of animals, and satisfactory physiological inferences from their structure, tend to prove that their purely instinctive actions are not the result of reflection or judgment, but as directly and certainly follow the excitement produced by external agents as do the most purely emotional or other involuntary actions in man. In all the higher animals we find more or less of the reasoning power superadded; but the exercise of this power is probably always connected with the instinctive propensities of the being, instead of having an independent operation, as in man. We have no reason to believe that a brute can ever bring its will to bear upon its own mind, so as to control its irregularities or direct the sequence of its ideas; and this appears to us a grand distinction between the intellect of man and that of the highest quadruped.

We have thus endeavoured to analyze the psychical as well as bodily conditions under which muscular contractions may be produced; and we shall now connect together these two classes of facts in such a manner as to lead to more particular inferences. Our object will be to determine what parts of the nervous system are severally concerned in the production of the voluntary and instinctive actions of the animal frame; and we think that this may be accomplished most certainly and readily by considering these actions in the order of their progressive complication. We have employed the term *instinctive*, here and elsewhere, to denote much more than is included under it by many writers: some have restricted it to one class of excited actions, some to another; but we think that it may be applied with the greatest propriety to designate all those changes in the muscular system which are immediately excited by impressions from without, which are not dependent upon the exercise of the will, though more or less capable of being controlled by it, and which, if acting alone, deprive the being of the character of a free agent. It is obvious, therefore, that these actions may be subdivided into several classes; and we think that the following arrangement of them will be satisfactory, both as regards the function they involve and the instruments by which they are effected.

1. Muscular motions directly excited by external stimuli applied to the organs which perform them, immediately connected with the functions of organic life, and incapable of being controlled, directed, or antagonized by the will. Such are the actions of the heart and muscles of the alimentary canal.

2. Muscular actions excited indirectly by impressions acting through the nervous system, but not of necessity producing sensation; less closely connected with the functions of organic life, though necessary for their *continued* performance; and generally capable of being, for a time at least, restrained or antagonized by the will. Such are the naturally associated movements of respiration, deglutition, &c.; the involuntary movements excited by application of artificial stimuli; the movement of the pupil, &c.

3. Muscular movements excited, still less directly, by the production of sensations which act upon the motor nerves without the intervention of reasoning processes; remotely connected with the maintenance of the functions of organic life, but generally having a particular tendency to the preservation of the system or the perpetuation of the race; and always capable of being controlled or directed by the will, when the latter is sufficiently powerful. Such are the involuntary movements excited by the special sensations, directed towards the acquirement of food, the construction of habitations, or the congress of the sexes; those concerned in balancing the body, and otherwise preserving it from suddenly impending injury; and those immediately connected with the excitement of the mental emotions; as also various acquired habitual movements. Each of these classes we shall now consider in detail.

I. Of the first we have but little to say, as it is not our intention to enter upon the old and, we had hoped, decided controversy regarding the source of the irritability of muscular fibre. The analogies we have drawn from the vegetable kingdom must be allowed some weight; and we can only stop to express our surprise and regret that Mr. Grainger should have so far suffered his usually discerning mind to be led astray by a favorite hypothesis as to suppose, not only that the contractions of the heart and alimentary canal are of an excito-motor character, the sympathetic nerve being their centre; but that the motions of plants may be "readily explained upon the principle of the reflex action." It certainly does appear to us a little strange that Mr. Grainger and other writers cannot allow contractility to be a property of living vegetable or animal tissue, as much as the capability of producing organic compounds or any other vital endowment. No vegetable physiologist of any intelligence now finds any difficulty in the "presumed necessity of sensibility in plants;" and no anatomist that we are acquainted with now imagines that the traces even of a nervous system can be discovered in them. It certainly behoves those who maintain that the irritability of muscular fibre is dependent on nervous energy to bring forward positive proof of it; as the *onus probandi* cannot be regarded as resting on those whom every analogy supports. Mr. G.'s deficiency in clear ideas on the subject is further shewn by his remark (true in itself) that "the first great step in the path of improvement (regarding our knowledge of the relations between nerve and muscle) was made by Haller, when, completing what was begun by Glisson, he succeeded in proving the distinctness of the muscular property, contractility, and of the nervous power, sensibility." If contractility be a distinct property of muscular fibre, where is the necessity for supposing, contrary to all observation and experiment, that the action of the heart depends upon nervous influence? With regard to the individual cases in which this application of muscular contractility is exercised in the living body, it may be observed that it is immediately connected in all with the performance of the organic functions, (to suspend which, even for a brief space, would be fatal;) and that this muscular apparatus forms a part of those additions to the essentially vital organs which are required by the conditions of animal existence.

II. The second class of instinctive actions cannot be dismissed so briefly, since to establish its real nature is the main purpose of the present article. We shall defer until its close what we have to say of the past history of the enquiry, concerning ourselves at present merely with the facts which it has disclosed, and the inferences which we think ourselves justly entitled to found upon them. The actions comprehended in this class are of a very diversified character; those originally belonging to it are probably all connected closely, either with the maintenance of the functions of organic life,—as the movements of deglutition with the supply of the digestive system, or those of respiration with the continued aëration of the blood; or with the preservation of the body from being injured by external agents, whose presence is usually recognized through the organs of common sensation,—as when a limb is involuntarily withdrawn from a flame, a pinch, a prick, &c.

All these subdivisions, which differ only in the tendency of the actions they respectively include, agree in the channel through which these actions are performed. In all it is necessary that the nervous communication from the part excited and the part exhibiting motion should be complete with the spinal cord,* and that the division of this organ, with

* Here and elsewhere we include in this term the medulla oblongata and the spinal origins of all the nerves. Similar views, expressed in a more general form, will of course apply to these parts of the ganglionic system of the Invertebrata which correspond with the cerebro-spinal axis in higher animals.

which the nerves conveying the impressions are connected, should be continuous with that from which the motor nerves arise. Frequently, and indeed generally, the *afferent* and *efferent* nerves are connected with the same segment of the cord; and, if this be entire, nothing more is necessary for the excitement of actions of this class by their appropriate stimuli. We shall now consider in some detail the character of each of the subordinate divisions to which we have adverted.

In the adult human being, the motions adapted to the ingestion and mastication of aliment, which were originally dependent on separate acts of the will, are probably performed subsequently through a shorter channel. The action of sucking in the infant, however, is from the first purely instinctive in its character, and belongs to the class we are now considering. The experiments provided for us by nature in the production of anencephalous monstrosities fully prove that the nervous connexion of the lips and respiratory organs with the spinal cord alone is quite sufficient for its execution; and the following experiments, performed under Mr. Grainger's direction, still more clearly reveal its nature.

"The brain was removed in a young puppy, which was then put to a large bitch, not the mother, but which was suckling at the time. The puppy, on touching the mamma, threw up its nose and moved the mouth, trying to get hold of the nipple; which, however, was too large. My friend and colleague, Mr. Barron, to whom I am much indebted for the performance of this and many other experiments, then moistened his finger with sugar and water, and put it into the mouth, when the puppy sucked, the tongue being wrapped around the finger. Mr. Barron also observed that the movements became more distinct when he pressed against the tongue.

"The brain was carefully removed in another puppy: the animal performed the same actions as in the last experiment, only that they were more vigorous; which was attributed to the hemorrhage being less in this than in the former instance. But the most remarkable fact was, that, as this puppy lay on its side sucking the finger, it pushed out its feet in the same manner as young pigs exert theirs against the sow's dugs." (P. 80.)

Our opinion as to the degree in which sensation is implied in this action must necessarily depend upon the view which we take of the functions of the spinal cord in general; and, though nothing in these experiments affects the question most at issue, yet the following interesting facts on the same subject must be allowed some weight.

"Nature, in the generation of the Marsupialia, displays, among the other extraordinary phenomena observed in those animals, several which are the exact counterpart of the actions of the anencephalous infant and of the animal deprived of its brain. In this interesting class of animals the fœtus quits the uterus at a very early period: in the Virginian Opossum, uterine gestation continues twenty-six days; and in the Kangaroo, according to the accurate observation of Mr. Owen, only thirty-nine days. The fœtus of the latter animal resembled an earth-worm in its colour and semi-transparent integument; adhered firmly to the point of the nipple; breathed strongly but slowly, and moved its fore-legs when disturbed. Its whole length, from the nose to the end of the tail, when stretched out, did not exceed one inch two lines. The brain in a mammary fœtus, one inch and a half in length, corresponds to the same organ in the human fœtus at the ninth week. Are not these the very actions of an anencephalous infant, or of an animal without its brain? The mammary fœtus breathes, moves its limbs *when touched*, and by the contraction of its mouth grasps the nipple. It is true it does not suck, because at this early period the muscular force is insufficient for such a continual action; and hence the beautiful provision of the compressor muscle described in the interesting memoir of Mr. Morgan; but it is certain that the lips, when they first are touched by the nipple, must contract upon and grasp it; and that subsequently the same action must incessantly be continued till, as the development proceeds, the fœtus becomes at times separated from the nipple. Can it be imagined that in this case there are sensation and volition in what can be proved anatomically to be a fœtus?" (Pp. 81, 82.)

The analysis of the actions next concerned in the supply of food to the digestive system is very important to the present enquiry. It is now generally allowed that the will has no direct power over the muscles concerned in the act of swallowing, and that an impression on the fauces is essential to the excitement of their actions. Until recently, however, it was believed that, though this action is constantly being performed during sleep or in profound coma, *sensation* is a necessary link in the chain of causation. In the act of deglutition, under ordinary circumstances, sensation accompanies the impression which acts as the real stimulus; but, as this action is performed equally well when the brain is removed, and when (as we shall presently shew) there is no sensation, we cannot regard it as *dependent* even on this primary mental condition. It was originally stated by Dr. Hall (*Memoirs*, p. 7,) that, when the food has once passed the fauces, its progress down the œsophagus is caused by the irritability of the muscles immediately excited by contact, as in the lower part of the alimentary canal. Dr. Reid's experiments have shewn, however, that the contraction of these muscles is occasioned, like the action of deglutition, by the impression propagated through their afferent nerves to the spine, and reflected by the efferent motor fibrils; and that the continuity of the nervous circle is therefore essential to it.* No one is conscious of any sensation accompanying the usual passage of food down his œsophagus; so that, as this action exhibits the necessary conditions in their simplest form, we can scarcely doubt that the movements of deglutition are excited by a similar influence. With regard to the nerves concerned in these actions there is much difference of opinion. By Mr. Mayo and Professor Müller it is believed that the glosso-pharyngeal is both a sensory and motor nerve; and Mr. Grainger regards it therefore as partly a sensory nerve, conveying impressions to the brain from the base of the tongue and fauces, and partly a true spinal nerve, exciting, by its incident and reflex action, the dilatation of the pharynx (by the contraction of the stylo-pharyngæus muscle,) consequent upon the contact of food with the fauces. Dr. Reid, on the other hand, regards it as only a sensory and excitor nerve; believing the pharyngeal and œsophageal branches of the par vagum to be the motor nerves of the muscles of this part. He found that section of the glosso-pharyngeal nerve by no means prevents deglutition, the stimulus being conveyed by other nerves; but that, when the pharyngeal branch of the par vagum is divided, the muscles are paralysed, and that small morsels only of the food can be forced into the œsophagus by the action of the tongue and flexion of the neck. The lower part of the œsophagus depends for its contractility upon the

* As we do not perceive that Dr. M. Hall has anywhere corrected the above misstatement, we cannot regard it as either just or generous on his part to say, as he has recently done, (*Lancet*, Feb. 17, p. 735,) "Dr. Reid's experiments confirm my own in every respect, and, I think, add nothing to what I had done." To Dr. Reid appears to us justly due the credit of having first established a clear case of *reflex action without sensation*, as one of the ordinary functions of the nervous system.

œsophageal branches of the par vagum, part of which seem to act as excitor and part as motor nerves. Dr. Reid very justly remarks, that we have here a satisfactory instance of the excitement of motions by simple impressions unaccompanied by sensations. "The food is propelled along the œsophagus without our consciousness and without our volition; and yet we have seen that, before the presence of ingesta in this tube can excite its muscular fibres to contract and propel their contents onwards, the same conditions of the nervous system are necessary as for the production of those sympathetic or instinctive actions which are not excited by mental acts."*

It was ascertained by Leuret and Lassaigne that the sphincter of the cardia is paralysed by division of the pneumogastric nerves; and the actions of this orifice, entirely independent of sensation, are therefore due to the maintenance of its nervous connexion with the spinal cord. The experiments of M. Brâchet tend to prove that the motions of the muscular parietes of the stomach, so essential to chymification, are also due to the influence propagated from the medulla oblongata, in obedience to the impressions conveyed to it, the par vagum being the channel of communication in each instance. Although the results of similar experiments performed by other physiologists have been of a different nature, we are inclined to believe that this is the true explanation; and we understand that the recent experiments of Dr. J. Reid dispose him to adopt a similar conclusion.† We can scarcely regard it as doubtful that, in the lower part of the alimentary canal, muscular action is altogether independent of nervous agency, and occurs in its simplest form, being excited by stimuli applied to the fibres themselves.

The foregoing is a brief sketch of the successive stages of the connexion between the animal powers and the most important of the organic functions. We have seen that, in man, the purely voluntary motions required to obtain and convey food to the mouth are followed by others which habit probably renders independent of the will, the whole process being in the infant a purely instinctive action; that these movements give place to others in which sensation even is not required, but in which the nervous system appears to act simply as a conductor; and that the object of these is to supply the stimulus to others immediately connected with the organic functions themselves, and not involving any vital property of a purely animal character. If we were to contrast these consecutive stages with those which a similar analysis of the actions of the lower animals would present to us, we should find that, as we descend the scale, we gradually lose the connexion of volition, next of sensation, and finally of any nervous system whatever, with the actions ministering to digestion; and that, in the simplest animals, as in plants, the necessary motions appear to result from mere excited irritability.

Some difficulty exists with regard to the character of the powers by

* Edinburgh Med. and Surg. Journal, No. xlix. p. 155.

† Two of his experiments already published countenance the above supposition, (*Op. cit.* p. 154;) and the distribution of the great nerve to the stomach in insects is so analogous to that of the par vagum in vertebrata as to afford some anatomical confirmation of it. We cannot, however, regard it as very improbable that the stimulus of innervation may be confined to the cardia, and that the muscular contraction there excited may be propagated *along* the stomach, as in the lower part of the alimentary canal.

which the other extremity of the alimentary canal is guarded; and we shall again look for assistance in comparative physiology. The formation of excretions is an act purely organic, and is performed by plants as well as animals. In the former the act of excretion is as constant as that of absorption; and in the lowest of the animal kingdom it would seem to be on the same footing, being as involuntary as the introduction of aliment; and both processes continue, with no observable intermission, during the life of the being. In more complex organisms, where the aliment is retained for the purposes of digestion, the discharge of excrementitious matter from the cavities provided for its reception is only occasional; and there is therefore a demand for a special set of associated movements to carry this purpose into effect, these movements being involuntary in their character, and excited by the quantity or stimulating quality of the contents of the reservoir. But, had volition no control over them, great inconveniences would arise: hence, sensation is excited in the perfect organism by the stimulus which would of itself produce the movements, in order that, by arousing the will, the otherwise involuntary motions may be to a certain extent restrained or directed. There can, we think, be little doubt that, from the experiments of Dr. M. Hall,* as well as from other considerations, that the associated movements by which the rectum and bladder are emptied correspond with those of deglutition, except in being capable of greater voluntary restraint or assistance; whilst the discharge of the contents of the vesiculæ seminales would seem to be completely automatic, and thus to bear a close resemblance to the action of deglutition. Here, again, we have evidence that the unbroken connexion of the nerves with the spinal cord is all that is requisite, and that sensation does not necessarily intervene in their production. These expellent actions are antagonized by the sphincters; and the question of the nature of their action is one of considerable difficulty. From an experiment of Dr. Hall's, (*Mem.* i. § 37,) as well as from the observed actions of anencephalous fœtuses, it would appear that the sphincter ani is entirely dependent upon the spinal cord, and that its contraction is of a simply automatic kind. On the other hand, the sphincter is certainly in part controlled by the will, and made to act in obedience to the warning given by sensation; and this voluntary power is frequently destroyed by injuries of the brain, when the spinal cord is healthy, and when the motions of respiration and deglutition are normally performed. We are inclined to believe that, in their moderate action, the expulsors and the sphincters balance one another as far as their excito-motor character is concerned; that, when the quantity or quality of the contents of the rectum produces an excessive stimulus to the former, their action predominates, unless the will is put in force to strengthen the resistance of the sphincter; and that, when the stimulus is deficient, the will must aid the expulsors, in order to overcome the excited contraction of the sphincter depending on the spinal cord alone. We are much mistaken if this view will not reconcile the difficulties under which Mr. Grainger labours on the subject; and at the same time we would refer our readers to his interesting discussion of it. A precisely similar view may be applied to the associated movements concerned in urination.

* Memoir, ii. § 172, &c.

The movements concerned in the function of respiration constitute a very interesting group, which we fully agree with Dr. Hall and Mr. Grainger in referring to the present class. That the medulla oblongata is not their centre, otherwise than by serving as the medium of communication between the afferent and efferent nerves, we are perfectly convinced. That the actions of deglutition and respiration, which are most essential to the maintenance of the organic functions, should be connected with a part of the cord most secure from injury, can excite no surprise; and, when this connexion is borne in mind, it affords a full explanation of the influence of the medulla oblongata upon the general vitality of the system. It is also to be recollected that, being the part of the spinal cord which is connected with the brain, its relations with the functions of that organ are necessarily of a very important character. No motions can be produced by mental influence, except through its intervention, and the communication of, probably, all the impressions by which sensation is produced takes place through its medium. Hence we have no difficulty in understanding its importance, without having recourse to any vague suppositions of its peculiar vitality.

What is the nature and channel of the stimuli to the ordinary movements of respiration is a question not yet fully decided. There can be no doubt that the spasmodic closure of the glottis, occasioned by the contact of foreign substances or of injurious gases, is due to an impression conveyed by the incident portion of the pneumo-gastric nerve, and reflected along its motor fibrils; and Dr. Reid's experiments seem to prove that the superior and inferior laryngeal act in these capacities respectively. It is believed by many physiologists, and by Dr. M. Hall and Mr. Grainger among the rest, that the impression produced upon the pulmonary extremities of the par vagum by the presence of carbonic acid or of venous blood in the lungs, is the ordinary stimulus to inspiration; and they rely partly upon the experiments of M. Brâchet in proof of this opinion, and partly upon the fact stated by Dr. Hall and Mr. Broughton, and confirmed by Dr. Reid, that compression of the nerves is followed by powerful respiratory movements. A great difficulty arises, however, from the fact that, after this nerve has been divided, the regular respiratory movements continue, although generally more slowly than usual. This is attributed by Brâchet to *custom;* by Dr. M. Hall to *volition;* and by Mr. Grainger to the continued conduction of the impression by the sympathetic nerve, which appears to be also the opinion of Dr. Reid. The first explanation we regard as entirely fallacious, since no habitual actions are excited without a stimulus. Dr. Hall's view is supported by a statement, which is certainly an important one if entirely correct, that respiration will not go on when both the cerebrum has been removed and the par vagum divided, though either operation may be performed singly without checking the movements. Dr. Reid, on the other hand, states that the motions continue after section of the nerves, even when all volition has been destroyed by a dose of prussic acid; and he has also proved that the anastomosis between the superior and inferior laryngeal nerves (to which we should otherwise have been inclined to attribute the effect, where the nerve, as is usual, is divided between their origins,) is not the means of sustaining them. We cannot pass by Mr. Grainger's experiments on this subject without noticing a serious objection to which his mode of performing them is liable. When unmutilated animals are placed in a limited quantity of air, they *gradually* become stupified by the accumulation of carbonic acid, and do not manifest the uneasiness which would be produced by the *sudden* diminution of the respiratory function in corresponding proportion. Hence, when animals whose par vagum has been divided are similarly circumstanced, it can excite no surprise that they should not manifest symptoms of greater disturbance. For the present we are compelled to incline to the last of the three hypotheses above mentioned; but we would suggest whether the cutaneous nerves in general may not in such cases, as in that of the first inspiration, conduct the stimulus to the respiratory movements. Dr. Hall has justly dwelt upon their importance to this action, and has adduced an instance in support of his view, which we shall quote as being of an interesting though not unique character.*

"My friend, Dr. Heming, witnessed an interesting fact in proof of this opinion. The infant, just born and covered by the bedclothes, did not breathe. Dr. Heming, after waiting a few seconds, proposed to himself to adopt some measure for this asphyxia, and lifted up the bedclothes. The contact of the cool atmosphere instantly excited an act of inspiration. This view of the subject is confirmed by some facts in pathology to be detailed shortly, and by some experiments." (*Memoirs*, p. 88.)

That all the actions hitherto described are dependent on the spinal cord and its nerves alone, has been abundantly proved by vivisectors, as well as by observation of the movements of anencephalous fœtuses. For some very interesting and judicious remarks on the latter subject, with which we fully coincide, and the length of which is the only obstacle to our extracting them, we refer to Mr. Grainger's Observations, p. 76, &c. Those of our readers, however, who have been accustomed to regard this part of the nervous system as endowed with sensibility, may not yet feel altogether satisfied that sensation is not a necessary link in the chain of excited actions. We think that the candid examination of the phenomena included in the next division, in which the organs usually regarded as voluntary will be shewn to be capable of similar excitement, should convince them that the indications usually regarded as establishing the existence of this property by no means warrant the inferences which have been drawn from them.

The movements we are now to consider are those of which the tendency is, either directly or indirectly, the protection and conservation of the body from external injuries; and for these the voluntary muscles are usually employed, although, in one of the most remarkable instances to which we shall allude—the action of the pupil—the will has not, except in a few extraordinary cases, any controlling power. That the aperture of the pupil is proportional, in a healthy eye, to the degree of light which impinges upon the retina, is a fact known from an early period. That it depends upon the sensibility of the retina has generally been believed, notwithstanding the cases, of no unfrequent occurrence, in which perfect amaurosis is accompanied by an active pupil. Mr. Mayo's experiments appeared to prove that the motion is under the influence of the third pair

* See a curious instance among the Selections from the Foreign Journals, in the present Number, p. 582.

of nerves, excited by a sensation communicated by the optic; and yet cases also occur (of which we have ourselves seen many,) in which complete paralysis of this nerve has been accompanied by an active pupil. The doctrine of excited actions seemed to us fully capable of reconciling these anomalies, and of serving, by its discriminative application, as a means of diagnosis; and we were accordingly well pleased to find that Mr. Grainger had taken a precisely similar view, and expressed it in his usual clear and simple manner.

"The optic nerve, like the common sentient nerves of the skin, contains two orders of fibres,—the true sensiferous, which are connected with the cerebral convolutions by the diverging fibres described by Spurzheim, and the incident fibres attached either to the grey matter of the optic tubercles or of the optic thalami. It is not possible to speak with certainty on this latter point, because the actual distinction in the use of the tubercles and thalami has never been determined by experiment; but, as Flourens remarks that the loss of the former does not destroy the motions of the iris, the incident fibres are probably connected with the latter. Now, those impressions made on the retina which produce vision are, it is certain, transmitted by the true sensiferous fibres to the cerebral convolutions; for, as Flourens has proved, the loss of the hemisphere, the other parts remaining intact, destroys in the animal the power of seeing; whilst it is as certain, from the experiments of Mr. Mayo, that the impressions which excite the actions of the iris are transmitted by those fibres which go either to the optic tubercles or to the thalami." (P. 72.)

"The existence of two orders of conductors existing in the optic nerve, and transmitting impressions either to the brain or to the optic tubercles or thalami, offers also a clue to those apparent anomalies in the state of the pupil which are so often observed in injuries of the brain producing coma, in apoplexy, and in amaurosis. In these affections, in all of which there is total blindness, the pupil is sometimes immoveable, whilst at other times it readily acts under the stimulus of light; and all this occurs as if there were no fixed laws regulating the actions of the iris. But, if a series of observations were instituted to determine these and other phenomena connected with the reflex action of the spinal cord, (and such an enquiry would be of great interest,) it would doubtless be determined that when, in compression of the brain, the iris retains its power, although there is a loss of vision, the cause of compression is confined to the percipient organs, the cerebral hemispheres; and that, when there is both a state of blindness and a fixed pupil, the mischief implicates the optic tubercles and thalami, as well as the hemisphere. The same remarks apply to cases of amaurosis depending on disease of the brain. Of course, in all of these cases, if the trunk of the nerve or retina is injured or diseased, the pupil will be fixed."* (P. 74.)

Here, then, we have another unequivocal instance of the production of reflected action through the spinal cord, without the necessary intervention of sensation.

One of the simplest instances of preservative tendency in movements of this class is afforded by the closure of the eyelid consequent upon the contact of a foreign substance with the tarsi, which Dr. Hall has shewn to occur when the functions of the brain are destroyed. In this and other instances sensation certainly follows the impression when the nervous system is entire; but just as we have shewn that the maintenance of the functions of organic life is remotely dependent upon actions, to the

* In a note Mr. Grainger has, however, complicated his explanation with what we consider the unphilosophical, because unnecessary, hypothesis, that the action of the pupil, through the excito-motor system, is stimulated by the *calorific* rays of the luminous object. We see no reason why light should not produce the requisite impression as well as heat.

excitement of which sensation is a necessary stimulus, so it would appear that, in these cases, its occurrence has a purpose no less obvious. For, as Mr. Grainger very justly remarks, "the accompanying pain is the best precaution that could be devised to prevent injurious effects, as it causes the animal to avoid in future all such sources of mischief." (P. 102.)

Our readers are probably already aware of the fact, first brought prominently forwards by the experiments of Flourens, that many of the higher Vertebrata will, after the removal of the cerebrum, execute most of their natural movements, when these are excited by their appropriate stimuli; and that the power of combining these movements appears to be lost when the cerebellum is also removed. The rabbit made the subject of this experiment by Magendie " cried when a hair of its whisker was plucked, or a strong acid applied to the nose, and endeavoured to remove with its paws any source of irritation, as it would do if it had not been mutilated." These and similar phenomena have been regarded as indicating the persistence of sensibility in the spinal cord. This conclusion has been greatly invalidated, however, by other experiments, which lead to an inference of an opposite character; but, although these experiments are very interesting, and the inferences drawn from them highly probable, we cannot regard them as yielding that satisfactory demonstration which pathological phenomena in the human subject afford. Some of the most striking of these experiments we shall quote from Mr. Grainger, by whom they appear to have been more discerningly executed than the similar ones detailed by Dr. M. Hall.

"The body of a salamander was cut into two pieces, so that the pelvis, with the hind legs and tail attached to it, was entirely separated, and the phenomena of the reflex power were carefully noticed. Some convulsive motions of the legs and tail, as in all other instances, took place. Upon pricking the feet, the limbs were freely moved, and also the tail; and, from the application of one stimulus, the motions were several times repeated, in consequence of the limbs at each movement coming in contact with the table. The effect of applying great heat,—a piece of lighted paper, for instance,—was yet more remarkable; for, upon touching one of the legs or tail, the pelvis and tail were forcibly moved: those parts seemed, in fact, to be writhing under the excess of suffering. In this case, as in every other in which the reflex action is excited, it was found that the extent of motion depended on the intensity of the stimulus; a combination of circumstances than which it is scarcely possible to conceive any more decisive proof that feeling still remained; in fact, without some powerful evidence to the contrary, no other conclusion could be formed.

"In another salamander the spinal cord was simply divided without any further injury being inflicted. Upon pricking the foot and tail, the same motions were produced as in the former experiment; and upon applying a lighted paper violent movements were excited in the tail and legs. All this seemed to indicate the persistence of sensation and volition, and yet it was found that the limbs which could be so violently excited by mechanical irritation and by heat remained motionless under the impulse of the will; so that when the creature made an effort to walk, which it frequently attempted, the hind legs and tail were, as in the case of the rabbit, kitten, &c. dragged along completely paralysed. Now, as the very idea of volition implies a perfect control over any muscle which an animal may wish to stimulate, this utter want of such power is the most decisive proof which could be adduced to shew that in this and similar cases the empire of the will was destroyed by the section of the spinal cord. But it may be thought that, although under these circumstances there was no longer any volition, sensation might still remain; a supposition, however, inconsistent with the fact that, in this same salamander, I found, by proceeding cautiously, so that the animal could not see the approach of the hand, that an entire hind leg could be cut off with a pair of scissors, without the creature moving or giving any other expression of suffering.'* (P. 57.)

In a similar experiment on a frog, it was remarked that the application of heat to one of the extremities, either anterior or posterior, excited a simultaneous movement in both; an effect quite different from that produced by similar treatment in a perfect animal, in which the injured limb only is retracted. The following experiment upon the green frog exhibits a marked correspondence with that of Magendie just alluded to.

"Upon irritating the cloaca in one of these animals which had been decapitated, the most violent motions were excited in the hind legs, and *repeated attempts were made by these limbs to remove the instrument* with which the cloaca was touched. This fact I have since repeatedly seen in the green and common frog, both when the head was removed and when the spinal cord was divided in the back: the same thing may readily be noticed in the common fly and other insects after decapitation. I have also observed that, if after having cut off the head in frogs, fire is applied to the fore part of the trunk, violent motions to remove the source of excitement are made." (P. 59.)

"In these experiments it was found that when the surface of the body was touched, as in the case of decapitated serpents, the headless trunk of the salamander was constantly turned in the direction of the irritation, and that when both sides of the trunk were simultaneously pinched, the body made violent movements, which, if the head remained attached, would be called struggles. It is likewise particularly worthy of observation, that in frogs there is a marked difference in the degree of excitability in the anterior and posterior limbs after decapitation; the latter, which are almost exclusively employed in swimming, displaying when pricked more forcible movements than the former." (P. 60.)

It must be recollected that the doctrine of the existence of sensibility in the spinal cord is founded only on vague inference from such experiments as those of Flourens, who was himself led by them to an opposite conclusion; and we think that the inference of its non-existence, founded upon the experiments we have just quoted, is at least as legitimate. We are, however, furnished with positive evidence that motions of this class may be excited without consciousness on the part of the individual, by the experiments which nature performs on the human subject; the effects of disease or injury replacing in these instances the scalpel of the vivisector.

We formerly alluded† to a case of paraplegia in which irritation of the foot produced its retraction, without consciousness or will on the part of the patient. This was at the time a solitary instance; but another case of a still more striking character was soon afterwards published;‡ and the prediction was hazarded that others would be speedily brought to light, when public attention should be called to the question at issue.

* We have heard it more than once enquired why the excessive stimulus of the division of the nerve did not produce the usual sympathetic movements in the case here related. It seems to have been forgotten, however, that, as the leg was cut off, the most definite and constant of these movements could not be shewn. But it may also be remarked, that, as there is a great deal of difference in the tendency of particular parts of the surface to receive and transmit the impressions which give rise to associated actions, (the most *sensible* parts not always being the most *impressible*,) so it appears that impressions made upon the peripheral extremities of the afferent nerves have more tendency to excite them than those made upon the divided extremity of a trunk.

† Vol. III., p. 38.

‡ Edinb. Med. and Surg. Journ., xlviii. p. 33.

This has been verified by the subsequent publication of four cases of a similar nature, of which we shall give the details. (See also p. 519.)

"I am indebted to a most intelligent pupil, Mr. W. F. Barlow, of Writtle, Essex, for the following interesting case:—'John Bright, aged nineteen, climbed up a walnut-tree, on the 1st of October, 1836, for the purpose of picking the fruit; and, when he had attained a very considerable height, slipped, and was precipitated to the ground. He was soon afterwards found in a cold and pulseless condition, with his lower extremities numb and motionless. There were obstinate constipation, which was overcome by strong purgatives, and retention of urine, which required the introduction of the catheter. The following was the condition of the patient three months after the accident: The lower half of his body and inferior extremities were entirely devoid of sensation, and they were not in the slightest degree under the influence of the will. Sometimes the patient had cold shiverings; and, whilst the muscles of that part of the body supplied with nervous energy from above the seat of injury, were observed to shake, those deriving their nerves from below that spot were perfectly motionless. Notwithstanding the anæsthæsia and the patient's inability to effect a single movement through the medium of volition, when the integuments of the legs were pinched, or more particularly when the sole of the foot was tickled, the extremities were retracted with considerable force. A little cold water dashed upon the surface produced the same effect, though there was no feeling of coldness. One leg was constantly in the flexed position, and, if straightened, immediately recovered it again. When the catheter was introduced, the penis was excited into a state of complete erection; an effect consequent upon the gliding of the instrument along the urethra; at the same time the legs were drawn up and a twitching of their muscles was very obvious.' The spinal marrow was found, post-mortem, to be nearly severed in the neck.

"I am indebted to Dr. Budd for a case of paraplegia, in which the most extraordinary and forcible movements of the limbs took place whenever the bowels were relieved. In a case recently detailed by Sir B. C. Brodie, bart., to the Royal Medical and Chirurgical Society, effects similar to those described by Mr. Barlow took place on passing the catheter; the patient being totally unconscious of the contact of the instrument and of its effects." (*Hall's Memoirs*, pp. 63-4.)

Why such phenomena are not oftener witnessed in old cases of paralysis, the following experiments of Mr. Grainger's afford a satisfactory explanation.

"A portion of the sciatic nerves was removed in a dog, and also in two rabbits: it was found in the dog that the power of exciting the muscles of the leg to contract, by pinching the sciatic nerve, was entirely lost at the expiration of eleven weeks, and, in the rabbits, the same result was observed at the end of five weeks. Thus, it is perceived that in a short time, if the muscles are entirely inactive, as they are in paralytic persons in whom no attempt is made to excite them, they lose all power of being stimulated through the medium of the nerves." (*Grainger*, pp. 96-7.)

By these data, therefore, we regard that as now demonstratively proved of which we formerly spoke as an admissible supposition, "that the changes in the spinal cord, not the sensations which in the natural state accompany them, are the causes of the sympathetic or excito-motory actions."*

III. Although from their greater similarity to voluntary movements, and from their dependence upon sensation for which the brain is required,

* British and Foreign Med. Review; Vol. III., p. 38.—The question has been frequently put to those who uphold the doctrine we are now advocating, *why* sensation should be so constantly associated with these changes, if not essential to produce motion. We might fairly object to any reasoning from final causes in a question of facts; but the objection is very easily answered. In many instances the production of sensations is the stimulus necessary for the excitement of other actions required for the continued maintenance of those in question. (*Edinb. Med. and Surg. Journal,* vol. xlviii.

(experimental investigation being thus forbidden,) there is greater difficulty in limiting the actions which we have placed in our third class, yet we think that there would be no obstacle to our strictly defining them, if we had those means of analyzing the exciting causes of the motions performed by the lower animals which we possess in the case of man. A characteristic feature in this, as in the other classes of instinctive actions, is the uniformity of their occurrence, especially when contrasted with the variability of those which depend upon intellectual processes. Thus, every species of animals has its own peculiar food, which is selected without hesitation when its presence is recognized by the excitement of a sensation; various muscular movements, sometimes of a very complex nature, are required for the purpose; and these are performed with a constancy which forbids us to suppose that they are the result of any acts of judgment or will. The movements excited by the sexual instinct are of the same kind; and not dissimilar do we regard those executed for the construction of a habitation, protection of the young, change of climate, &c. Now, it has been said that in all these cases an instinctive propensity excites a desire which occasions the will to act; but we would ask, upon what evidence? It is inferred from the analogy of human actions; but this is not a safe ground of argument, unless the sources of those actions be carefully analyzed; and we think it will appear, from the preceding sketch, that the analogy is really on the side of their involuntary character. In man these propensities are never (except when morbidly excited) the immediate springs of action; they only produce movements by stimulating the intellectual faculties to provide means for their gratification; and in this respect they rank with ordinary emotions. But in man, when reason is undeveloped or dormant, these instinctive propensities manifest themselves in full force; and their action is then similar to that of an emotion predominating over the will, and, as we have shown, independent of it.

We see no reason why the special sensations (which are not concerned in the second class,) should not excite motions as directly as those impressions which appear to act through the spinal system alone; and various facts tend to support such a conjecture. Although, in adult man, the instincts which minister to the supply of the organic functions are entirely superseded by volition, to which they simply act as a stimulus, there are many motions destined to the preservation of the body from danger, which nature has wisely rendered independent of his uncertain and capricious will. The case to which we have formerly alluded* affords us an apt illustration: "the eyelids, although resisting the influence of the will, closed involuntarily on the sudden approach of a body towards the eye, or on the application of a strong light." This motion, therefore, is evidently as direct a result of the visual sensation as is that produced by tickling the edge of the tarsi in a case of anæsthæsia of the mere impresp. 38.) In other cases it contributes to enjoyment, (as in suction, ejaculatio seminis, &c.;) affords premonition of danger, or excitement to supplementary actions, (as in respiration;) gives warning against inconvenience, (as in the excretory functions.) In this last case the sensations are often excited, not to produce the associated actions necessary for the excretion, but actually to make the will set up the antagonizing action of the sphincters. (See p. 510.)

* Vol. IV. p. 500.

sion. The ordinary motions destined to preserve equilibrium are in like manner, as has been well shown by Dr. Symonds,* the result of sensations only, without the influence of volition; although there is no doubt that, in man, they are greatly modified by education.

Amongst the most remarkable operations of this connexion between sensation and motion in the human being, are the actions which result from *imitation* and *habit*. The former is a propensity which is capable, like others, of being morbidly excited and of overcoming the power of volition, so as to lead to the most extravagant actions; but it has a remarkable effect in producing involuntary actions when the mind is directed towards other objects, even without being itself more than naturally developed. In the article referred to, Dr. Symonds's remarks, "Any set of muscles may acquire particular actions and assemblages of actions by passive imitation only; and to such a degree, indeed, that desire is often vainly employed in opposition to this principle. One person yawns, or sighs, or laughs, because another does; a child or susceptible female, if frequently in company with a person who winks, or stammers, or faulters in his gait, will fall into similar habits, notwithstanding there may be a variety of inducements for attempting to avoid them."

The influence of habit in reducing to this class actions which were originally of a purely voluntary character is extremely remarkable; and we cannot help wondering that several able metaphysicians should so long have maintained that the will is still concerned in each movement. If it be true, however, as we have already argued, that muscular contractions originally resulting from a volition remotely excited by common sensation, may in time be excited by an impression only, there can surely be no reason for refusing to the special sensations a similar power; and it appears much more philosophical to suppose that a train of habitual actions, once started by the will, should be continued by the stimulus of sensations only, whilst the attention of the mind is stedfastly directed towards other objects, than that the intellect should be in a state of constant vibration between the two processes. It is perfectly true that the will can at once check these actions, but only when directed with that specific purpose; but, if this does not interpose, we are of opinion that the train once started will continue to run on as long as the required stimulus is supplied, and the organs of sensation remain in a direction or condition to receive it. Such actions may be excited by internal as well as external sensations, or by the recall of either by the aid of memory.†

It appears to us not improbable that the locomotive actions of man and the higher animals, considered singly, are of this character. In many of the inferior classes, probably in all below the terrestrial vertebrata, these actions are from the first purely instinctive; and the remarkable perfection and continuance of their performance by insulated segments in the articulated classes would show that they are not in them

* On the Relations between Mind and Muscle. — (West of England Journal, vol. i. p. 169.)

† It will be seen that on this point we adopt the opinions of Hartley, in opposition to those of Dugald Stewart, and others. The *secondarily automatic* actions of the former writer correspond precisely with our *habitual* motions excited by sensation only. For a full statement of the facts and arguments on each side, see Rees's Cyclopædia, Art. *Mental Philosophy*.

dissimilar to the movements of respiration in birds or quadrupeds. In those species, however, of which the young are born in a state incapable of maintaining their own existence, and dependent therefore on their parents, the locomotive apparatus has to undergo an education not only for the adaptation of single movements to particular purposes, but for the combination of them into the complex associated actions of walking, flying, &c. For this combination the existence of the cerebellum appears to be necessary; and it is then impossible to say what degree of sensation may remain. The following experiments, however, show that the part of the foot which habitually touches the ground is peculiarly susceptible of impressions by which propulsive motions are excited.

"In a rabbit about three parts grown, the cord was laid bare in the middle of the back, and a portion of it cut out. The lower part of the body and the hind legs were immediately paralysed, the limbs were laid motionless on the table, and the animal had no power of moving them; a fact which become very striking when the rabbit made an attempt to walk; for then, notwithstanding the fore legs freely acted, and thus dragged along the body, the hind legs were motionless; volition, although in full operation, had no effect upon them. I then, with a fine needle, pricked the skin covering the under part of the heel; when instantly *the toes became extended, the heel was raised and both legs were forcibly thrown backwards.* This experiment was repeated on several other rabbits, in kittens, and in puppies, and constantly with the same result; the combined and successive motions of the toes, the foot, and the leg, were seen; but, in the kitten, the irritation to produce the effect required to be made not on the heel, as in the rabbit, but nearer the toes. In most of these instances it was also found that only the leg belonging to the foot which was irritated was thrown backwards.

"In a similar experiment, performed by Mr. Mayo, it is stated that, when the foot was irritated, the movement of the limb was exactly similar to that which the animal would make if in indisputed possession of its sensation. In order to ascertain the correctness of this statement, I pricked the hind foot in a rabbit, the cord of which was entire, when the animal moved the limb to avoid the irritation; but the motion was totally different from that which occurred in the preceding experiment. Upon dividing the cord, and pricking the under part of the foot, a most violent motion was excited, and both legs were thrown back. Those gentlemen who were present were particularly struck with the difference of the movements in this rabbit before and after the division of the spinal cord. I found, in this and other similar experiments, that when the thin skin of the leg was touched after the section of the cord, a very slight contraction was produced, and that it was only when the under part of the foot, which in progression strikes on the ground was irritated, that the remarkable and combined action of both legs was produced." (*Grainger*, p. 54.)

A very interesting confirmation of this view is afforded by the following remarkable case; and we ourselves have seen a similar one.

"A girl, about fifteen years of age, who was a patient of Mr. Crosse, at the Norfolk and Norwich Hospital, a few years since, was affected with angular curvature of the spine, producing insensibility and paralysis of the lower extremities. On tickling *the soles of her feet*, which as an experiment was often done, the legs were immediately slightly retracted, although the patient said she felt nothing; it was further remarked that, on touching the *other parts of the feet* or *the legs* in the same manner, no effect was produced." (*Grainger*, p. 94.)

A case has been mentioned by Mr. Travers, in which the os frontis was driven in by a fall, and a considerable portion of the brain extruded at the wound of the scalp; yet the boy, "although utterly deprived of consciousness, made obvious but unavailing efforts to aid the surgeon in getting him into bed, and thrust his arm mechanically into the sleeve of a clean shirt, after his hand had been placed in the opening of the sleeve."

The following observations of Mr. Grainger's, with which we must take our leave of this part of the subject, we regard as worthy of attention, although we are not yet prepared to coincide with them.

"When, in walking, the foot strikes the ground, if we pay any attention at all to what takes place, we are only conscious of the sensation which is excited; the motions of the limb are overlooked, or it is concluded that they are purely voluntary: and so it is in the case of deglutition; a sensation is produced by the contact of the food, and, until lately, it was thought that the motions consequent upon that contact were voluntary. It is then, I think, most probable that, in progression, an impression is made on the incident nerves of the foot, by which the reflex power of the spinal cord is excited to action, and the combined and required motions are produced. This explanation does not in any degree exclude the idea of due and proper control being exerted over the limbs in walking, flying, swimming, &c., inasmuch as there are proper nerves, the cerebral, going to every voluntary muscle, by which the animal can at will direct, quicken, or give increased vigour to the motions; it is even probable that a special apparatus is provided for the purpose of enabling the animal altogether to prevent or stop the excited actions. It is also evident that these excited actions of the legs must be much more perfect and influential in the lower than in the higher animals, exactly in proportion to the relative development of the spinal cord and brain; so that, whilst in some of the lowest tribes progression is almost entirely a spinal action, in the mammalia, and above all in man, the central influence is greatly exerted. The muscles called voluntary are then probably all susceptible, under different circumstances and in varying degrees, of being stimulated by the brain, through the medium of volition, and by the spinal cord through the means of the reflex power." (*Grainger*, p. 150.)

We have now analyzed the characters of purely instinctive actions, and traced them to the parts of the nervous system on which we regard them as respectively dependent. We are well aware that the extended application of the term *instinct* which we have employed may not suit the ideas of many of our readers: it is, however, one which has been used by several of those who have most successfully investigated the nature of the actions thus included in it, and who think, as we do, that no distinct line of demarcation can be drawn between any of those actions of living beings which are immediately prompted by external stimuli, and in which no purely mental acts are involved.*

Of the functions of the great sympathetic nerve, which Mr. Grainger regards as a collection of systems of ganglia with their respective incident and reflex fibres, by the agency of which are produced the contraction of all the organs which are in a more especial manner supplied by it, we have little to say in addition to the observations we have made in this and in former articles on the influence of the nervous system on the functions of organic life. "If," says Mr. G., "it be admitted that any nervous power at all is required to effect those processes which take place in the fœtus—circulation, secretion, and nutrition,—(and it is almost impossible to arrive at any other conclusion,) it is certain that it is the agency of the great sympathetic, and not that of the cerebro-spinal system which is necessary." Now, we still maintain that it is incumbent on those who uphold the necessity of this nervous power to prove it definitively, since all analogy leads to an opposite conclusion. We regard the capability of

* See Kirby, Bridgwater Treatise; ch. xviii. Virey, Histoire de l'Instinct des Animaux; Dubois sur l'Instinct, Mem. de l'Acad. Royale de Médecine, tom. ii.

separating from the blood a peculiar secretion as a peculiar property inherent in the glandular membrane, as contractility is the inherent property of muscular fibre: and just as the peculiar arrangement of the excitable and contractile tissues in animals requires a nervous system to act as a conductor between them, and to blend their actions, independently of any production of sensation or influence of the mind, so do the complicated and highly specialized organic functions of animals require to be harmonized and kept in sympathy with each other by some mode of communication more direct and certain than that afforded by the circulating system. These functions also require to be placed in relation with the purely animal system, and especially to receive an influence from certain emotional conditions of the mind. This is unquestionably effected by the communications of the sympathetic or visceral with the symmetrical system, which increase in number as we ascend the animal scale.

We think that we have thus found quite enough to be accomplished by this nervous apparatus, which has been so unfortunate in the number and variety of offices with which it has been charged; and we hold that these functions are all that rigorous inferences from observation and experiment can lead us to attribute to it. If we regard the hypothesis of the dependence of the organic functions on nervous influence communicated from the great sympathetic, as wanting in the characters of a legitimate theory, we should express ourselves in still stronger language in reference to Dr. Hall's strange notion of the uses of the ganglia upon the sensory roots of the spinal nerves, which is unsupported by any but the vaguest analogies and is controverted by numerous facts.*

In order to account for the connexion between certain impressions upon the sensory organs and their resultant motions, many physiologists have endeavoured to establish a peculiar anatomical relation between the roots of those nerves which convey the impressions to the central sensorium and those which transmit the stimulus to the muscles. Our readers must be well aware that to establish this relation in the case of the respiratory nerves was one of Sir C. Bell's favorite objects; and that the principle which he thus assumed led him to infer the existence of a particular tract in the spinal cord, from which these nerves, or at least that portion of them subservient to the function, derived their origin. This doctrine was strenuously resisted by those physiologists who were not so far dazzled by the brilliancy of Sir C. Bell's other discoveries as to admit readily whatever he should advance; and it received what we cannot but regard as a very satisfactory refutation from the pen of Dr. Alison, to whose paper on Sympathy we shall presently have occasion to refer more particularly. In fact, as it has been more than once urged, "Sir C. Bell's attention appears to have been so entirely absorbed by those connected with the respiratory function, that he seems to have forgotten that there are other extensive associated and sympathetic movements of the muscles of the body, besides those which he has so beautifully illustrated; for it is obvious that, if a particular tract of the spinal cord is necessary to carry on

* Of these, one will at present suffice. In fishes, no ganglia exist upon the posterior roots of the spinal nerves; yet nutrition of the tissues is performed without them. As the scaly covering of fishes must greatly interfere with general sensibility, we do not consider that the supposition of the connexion of the ganglia with sensation is invalidated by this fact.

the respiratory movements, there ought also to have been a defecatory tract, a urinary tract, and so on, to carry on the other sympathetic movements in which a number of distant muscles are engaged in simultaneous action. If the other associated movements can go on without it being necessary that the nerves supplying the muscles concerned in them should come from particular tracts of the spinal cord, then surely there can be no necessity for this in the case of the respiratory nerves."* We are far, however, from wishing to argue against the principle of referring to structural connexion, wherever we can do so, the connected functions of different nerves. A very large proportion of the associated movements excited by impressions conveyed to the spinal cord are stimulated through the motor nerves arising from the same segment; and this holds good not only with regard to those usually regarded as spinal nerves, but to those which take their origin within the cranium. The supposition that there is an organic or structural connexion of some kind between nerves of apparently distant origin whose functions are associated together, appears to us therefore by no means absurd or even deficient in probability; and knowing as we do how many different functions are performed by the tubuli of any one nervous trunk, and how difficult it is to trace these far into the central organs, we are not surprised that it should have been hitherto difficult or even impossible to trace such connexion by anatomical investigation.†

* Dr. J. Reid, in Edinburgh Med. and Surg. Journal, vol. xlix. p. 173.

† We are well aware that the view here expressed is opposed to that which was maintained by Dr. Alison, in his paper on Sympathetic Actions, already referred to, and which this distinguished physiologist still holds. He regards Whytt as having demonstrated that these movements are the result of the excitement of particular *sensations* by which they are stimulated ; and endeavours to prove the fallacy of any attempt to explain them by connexion of nervous structure. Now, Whytt's object was to show that all sympathetic actions (in which he includes the motions of the heart and alimentary canal) are produced, not by *direct* nervous or other structural connexions, but by the excitement of the "sentient principle," which is intermediate between the application of the stimulus and the reflexion of it, and for which it was obviously necessary that there should be communication with the *central* organs of the nervous system. How far Whytt implied the existence of *sensation* by the use of this term, we shall presently enquire, (see p. 524;) but it seems to us that the action of stimuli through the *central organs,* rather than by any more *direct* channel of communication, is the main point of his argument. Now, there would seem no reason why particular motions should not be associated with particular *impressions* as well as with particular *sensations;* since it is obvious that, to produce a similar sensation, a similar impression must be made; and a difference of sensation necessarily involves the idea of a difference in the impression. Thus, impression *a* produces
sensation A, with which is associated
motion *α*;
and, in like manner,
impression *b* produces
sensation B, with which is associated
motion *β*.
We cannot see, therefore, why the production of motions may not be regarded as connected with the *impression* as well as with the *sensation.* That sensation always follows the impression in the normal condition of the system, we well know, and can readily account for; but this does not prove that the motion is associated with it; and, in the instance of the action of the pupil, we have abundant evidence, supplied by pathology, that the sensation may be *entirely* abolished, and the sympathetic or associated action yet be excited. It is obvious, therefore, that such views are in no degree opposed to the doctrine of continuity of structure between the afferent and efferent nerves, established through the central organs of the nervous system.

The views which we have already stated regarding the nature of the changes performed by the spinal cord, must have rendered it evident that we regard sensibility, and the capability of exciting voluntary motive action, as confined to the brain. It must be recollected that the sensibility thus attributed to the brain is a very different thing from the (so-called) sensibility of the external organs of the body. When we say that sensibility is located in the brain, we mean that external impressions must be communicated to that organ before the mind can become conscious of them; whilst if we say (as is inaccurately, though frequently done,) that the skin or other tissue is sensible, we mean that it is capable of being impressed by an external agent in such a manner that a sensation is excited in the mind, which is referred by it to the spot where the original impression was, or seemed to be, made.* We can thus readily explain the fact, proved by the experiments of Flourens, Fodera, and others, and by numerous pathological observations, that injury of the substance of the brain itself does not give rise to painful sensations; for it is only in obedience to impressions conveyed through a particular channel that such sensations are excited; and there is no more reason why lacerations or wounds of the substance of the brain should produce pain than that the exposure of the roots of the optic nerve to luminous objects should give rise to visual sensations. A very similar explanation will account for the non-production of motion by stimulants applied to the brain itself, and not directly affecting the medulla oblongata. All motions are excited by changes in the spinal cord, from which the motor nerves really arise: the ordinary office of the brain is to produce some of those changes. We can imitate, by chemical or mechanical stimuli applied to the cord, the effects of the stimulus which it ordinarily receives from the brain or from external impressions; but we cannot imitate those changes in the brain itself which give rise to those of the spinal cord.

We must now bring our review to a close by retracing, as briefly as is consistent with clearness and equity, the successive advances towards what we regard the correct opinions on the points now under discussion. The first reference with which we are acquainted to the doctrine of the transmission and reflexion of an impression through the nervous system appears in Glisson's remarkable work, "Tractatus de Ventriculo," (p. 172,) published in 1677; and, though the motions thus occasioned are not clearly expressed, and the central organs of the nervous system, not referred to in their production, it is evident that this discerning writer separated them, on the one hand, from the effects of simple irritability of muscular fibre, (which he was the first to regard as a property of that tissue independent of sensibility,) and from voluntary motion on the other.

We find, however, in the "Essay on the Vital and other involuntary Motions of Animals," published by Whytt, in 1751, a much fuller, and in most respects more correct account of these phenomena; and, as the opinions of this sagacious author are too little known to the present generation, and, owing to the misinterpretation of his peculiar phrase-

* This distinction will at once be made apparent by adverting to the *sensations* referred to the extremities of a nerve, when it is on some part of the trunk that the *impression* is really made.

ology, have been little comprehended, we shall detain our readers for a brief space whilst we set them in what we regard as their true light. It is well known that Whytt strongly opposed the doctrine of Glisson and Haller regarding the independent contractility of muscular fibre; and the object of his essay is to show that the vital motions,—that is to say, the muscular contractions immediately concerned in maintaining the organic functions,—are all dependent upon a *stimulus* conveyed from the periphery to the central organs of the nervous system, by which a motive influence is there originated. This motive influence he regarded as due to the *sentient principle*, which he believed to be a part of the soul, and to be diffused through every part of the body. Of the nature of this sentient principle, as elsewhere limited by himself, we shall presently speak. Dr. Whytt describes three kinds of muscular contraction, *natural*, *voluntary*, and *involuntary*. The *natural* contraction he explains to be the "constant contraction of the sphincters, and the tension of such muscles as are balanced by antagonists," (pp. 12, 13;)* and he points out the dependence of this power upon the nervous system, and at the same time its partial subordination to the will. It is evident that this "natural contraction" precisely corresponds with the "tone" of Dr. M. Hall; and, whilst we willingly yield to the latter physiologist the credit of having limited this operation of nervous power to the spinal cord, we cannot admit his originality in specifying its character.

Of the *evident* contractions he says, (p. 15,) "As often as the influence of the nerves is determined into the muscles, so as to operate more powerfully on them, they are excited into stronger contractions, which are not natural, and therefore may be called violent. This extraordinary determination of the nervous influence may be owing either to the power of the will or to a stimulus." The former are, of course, *voluntary* actions; and the *involuntary* motions he at first describes as arising from a stimulus applied to the muscles, which immediately excites them to contract themselves. This contraction he attributes to nervous energy; and observes that "the effects of different stimuli depend very much upon the peculiar constitution of the nerves and fibres to which they are applied." (P. 18.) He subsequently, however, extends the application of this principle, by showing that the stimulus is not always applied to the muscle itself, as in the case of the motions of the iris and those of respiration; and he argues that the sympathy in these cases cannot be explained by the connexion of the nerves themselves, but only by the common terminations in the brain. He maintains that the contractions of the heart and alimentary canal are altogether involuntary, because the stimulus is applied to the muscles themselves; and points out that, where the stimulus is conveyed from a distant part, as in the actions of respiration, defecation, &c., the will has the power of controlling the movements in the inverse proportion of the strength of the stimulus. (pp. 200-1.) The motions of the iris are mentioned as an exception to this principle, because "the action of light upon the very sensible retina affects the mind so strongly that we cannot, by any power of the will, prevent the contraction of the pupil." (P. 202.) After discussing the motions connected with the organic functions, Dr. W. passes on to those

* Whytt on the Vital and other involuntary Motions. Edinburgh, 1751.

designed for the preservation of the body; and instances the retraction of the leg owing to the fall of a spark or drop of boiling water on the foot, and the contraction of the muscles of the trunk occasioned by tickling the sides, as proofs that the stimulus does not produce motion by "sympathy of nerves and continuity of membranes," but is conveyed to the mind, which, by an involuntary action, endeavours to get rid of a source of disagreeable irritation. He argues also, from these facts, that in no case are the muscular fibres called into action by direct stimulation, as Haller maintained. The last chapter of his work is devoted to the consideration of the motions of animals after death, which he distinctly attributes to the same *sentient principle,* or soul, which produced them during life.

It will be seen, therefore, that Whytt had a very distinct and comprehensive notion of the character of the involuntary muscular actions included in the three classes we have formerly specified; and that, though his erroneous ideas of the contractility of the muscular fibre prevented him from attributing those composing our first division to their right sources, yet that he clearly distinguishes them from such as are excited by a stimulus applied to a distant part. But it will be said by those who are disposed to depreciate the value of his writings, that Whytt absurdly attributed all these motions alike to the operation of a "sentient principle," which he identifies with the soul. This would, however, be giving a very unfair, because incomplete, representation of his opinions; since no one can attentively read the essay referred to without perceiving that the writer uses the term in a far different sense from that which we should now attribute to it.* He clearly separates the *sentient* from the *rational* principle of the soul, and shows that, as far as the latter is concerned, the individual acts voluntarily, or is a free agent. Dr. Whytt's ideas of the universal necessity of nervous agency for muscular contraction, required that this sentient principle should be regarded as existing not only in animals recently dead, but in the separated parts of those which remain alive; and for his ingenious mode of explaining the simultaneous divisibility and unity of the soul, we must refer to his Essay, (pp. 382-3.) By Dr. Alison, and other modern physiologists, Whytt has been regarded as using the term "sentient principle" synonymously with what we now call "sensation." But we think that a candid examination of his essay will show that the term "excitability" more nearly expresses his meaning; for he very plainly shows that the operation of the *sentient principle* does not always imply the mental change which we now understand by *sensation.* The gentle and uniform stimuli to the vital actions he regards as operating without *consciousness* either of the presence of the stimulus or of any mental act excited by it, (pp. 292-303;) and it is evident therefore that his ideas, put into the phraseology of the present day, would be simply expressed by saying that such ordinary and gentle stimuli act by producing *impressions* only, whilst stronger ones, by which unusual motions are excited, produce *sensations* also. How nearly Dr.

* In illustration of this phraseology, we may refer to a curious contemporary treatise on Animal and Vegetable Propagation, published in 1752. The author, Dr. Parsons (like Whytt, a Stahlian,) speaks of the "animating principle" of plants, and of the "animating and moving principle" of animals, as forms of *the soul.*

Whytt thus approached to the doctrines we have been maintaining, we need scarcely stop to point out.

That Whytt was not ignorant of the *independent* functions of the spinal cord, is evident from the following passage: "Wheresoever, in the present argument, I have spoken of nerves being derived from the brain or cerebellum, I desire to be understood as including the spinal marrow, so far as it can be reckoned a continuation of the medullary substance of these parts, or to agree with them in its structure and use; for the spinal marrow does not seem altogether derived from the brain and cerebellum, but probably prepares a fluid itself; whence it is enabled to keep up the vital and other motions for several months in a tortoise, after the head is cut off." (P. 341.) If we couple this statement with Whytt's general proposition, that all the vital and involuntary motions proceed from a stimulus, (p. 325,) we can scarcely fail to recognize the doctrine of the "reflex function."

That our statement of Whytt's doctrines has not been warped by the desire of making them appear as conformable as possible to the present state of opinion on these subjects, we think that no one will deny, who candidly examines the Essay we have alluded to, as well as the preface to his work on Nervous Diseases. But we are enabled to produce stronger testimony in their favour from the writings of his eminent successor Dr. Cullen, who adopted, with some modification, his views on the influence of the nervous system, and whose phraseology is sufficiently like that of more modern writers to leave no doubt as to his meaning. Dr. Cullen's opinions with regard to the agency of the nervous system in producing muscular contraction were intermediate between those of Whytt and Haller, as will be seen from the following passage:*—"Though the muscular fibres of the heart be endowed with a certain degree of inherent power, or *vis insita,* they are still, for such as is necessary to the motion of the blood, very constantly dependent on a nervous power sent into them from the brain. At least this is evident, that there are certain powers acting primarily, and perhaps only in the brain, which influence and variously modify the action of the heart. I suppose, therefore, a force very constantly during life exerted in the brain, with respect to the moving fibres of the heart, as well as of every part of the body;"—and he then goes on to adduce, in support of this opinion, various facts illustrative of the influence of the different states of the nervous system upon the heart's action, which, in his opinion, prove the *partial* dependence, at least, of the latter upon the supply of the "Energy of the Brain."

It will readily be seen, therefore, that Dr. Cullen admitted some modification of Whytt's views, in accordance with the proofs adduced by Haller of the independent irritability of muscular fibre; but that he still regarded the influence of the brain as concerned in the production of all muscular contractions. The brain, however, he defines as including "the whole medullary substance within the cranium and vertebral canal." (*Physiology*, p. 102.) Its action he speaks of as being excited by the following causes:—1. By volition, when "we will the motions, as means to an end." 2. By the emotions or passions. 3. By the disposition of

* Thomson's Life of Cullen, p. 296.

human nature to imitation. "This imitation is sometimes involuntary; often without consciousness." 4. By appetites or desires directed to certain external objects, and arising from sensation without any reasoning directed to an end. "These motions are evidently instinctive, and strictly connected with the desire that excited them." 5. By certain propensities or desires to remove an uneasy or painful sensation; the motions thus excited not being directed to any external object, but confined to the body itself. "Very often the stimulus to these propensities (as in sneezing, vomiting, &c.) is irresistible; and, unless the peculiar stimulus is present, the motions cannot be produced by volition." 6. "By certain *internal impressions* arising from the exercise of the functions of the body itself, which produce no *sensation*, nor produce motions of which we are conscious, except when exercised in an unusual manner: such are the motions of the heart and arteries, of *the organs of respiration*, of the stomach and intestines, and perhaps of many other parts." 7. "By various occasional *impressions* of *external* bodies, and by various occasional states of the system or of its particular parts, which excite motions not only in the parts to which the impressions are immediately applied, but also in distant parts, on which they can operate only by the intervention of the brain. Some of these causes operate with, others without, *sensation or volition.*" (*Physiology*, pp. 105-111.) Now, though Dr. Cullen erred with Dr. Whytt in placing the heart's action and the peristaltic motion of the intestines on the same footing with the movements of respiration and other associated muscular contractions, this does not diminish the importance of the fact that he recognized in the nervous system a power of respondence to *impressions* only, without the necessary excitement of sensation. And as Cullen professedly adopted the opinions of Whytt on this subject, we cannot hesitate in accepting his expression of them as confirmatory of the view we have already given of the doctrines of the latter, and to which we had been led by the careful perusal of his own works alone. We cannot but regard the last passage which we have quoted as containing, under its sixth and seventh heads, all the essentials of the doctrine of the "reflex function," broadly and distinctly stated in language which cannot be questioned; and, had Cullen pushed his analysis further, he would probably have been led, by Whytt's experiments, to the conclusion that the spinal cord is the only part of "the brain" specially connected with this property. We have no record, however, of his having attempted to specialize the functions of the different parts of the central organs of the nervous system: this was accomplished by later physiologists.*

When speaking of *Sensations*, Dr. Cullen divides them into Sensations

* It is a little remarkable that Cullen should have adduced, among other illustrations, a topic which has since given rise to much controversy, and on which the latest and most unexceptionable experiments seem to lead back to the opinion which he expressed. "The action of the stomach," according to Dr. C.† "is not so constant as that of the heart and organs of respiration, but it is pretty constant. . . . That the stomach requires the constant energy of the brain is proved by its immediately becoming paralytic when its nerves are destroyed. This effect was commonly found to occur in the experiments made upon the heart by cutting the par vagum: the appetite was destroyed, the contents of the stomach stagnated, and were variously corrupted."

† Thomson's Life, p. 298.

of Impressions derived from without through the sensory organs; and Sensations of Consciousness, "comprehending a variety of feelings in the mind, of which we are conscious, but which we are not able to trace to the impression of bodies external to the nervous system." Under the latter head he includes not only the consciousness of mental operations, but what are now generally called *internal sensations*, arising out of particular states of body, and connected with the organic functions. With this explanation we shall quote the following remarkable passage, which seems to us to comprehend what is essential in the opinions we have expressed on habitually excited actions, and at the same time to correspond in fact, though not in appearance, with the statements of Whytt on the same subject—"Actions which at first produce a sensation of consciousness, as being accompanied with volition, come by repetition to be performed *without any sensation;* or they produce it only when they are performed with uneasiness, pain, or unusual force. When voluntary actions are seldom repeated, the mind is conscious, as often as they occur, of its volition producing them; but if they are very frequently repeated, the mind will at length nearly, or altogether, lose the consciousness of the exercise of its volition; and a motion of this kind will now be excited by external agents, or by any state of the body that formerly occasioned a volition of the mind, without any consciousness of the will being concerned in producing the motion, or at least without any subsequent recollection of the will having been exercised." (*Life, &c.* p. 321.)

The next writer to whom we shall refer is John Hunter, whose Croonian Lecture on Muscular Motion, delivered to the Royal Society in 1776, but just published for the first time, contains many valuable observations relating to our present subject. We do not, of course, refer to these as disparaging the merit of more recent authors, who had no direct means of becoming acquainted with them; but rather as illustrating the gradual progress of opinion on the influence and action of the nervous system: it is right, however, that we should state that many of Hunter's views were expressly included in the writings of his successor in the Lectureship, Sir G. Blane, who, as we learn from good authority, was present at these lectures, and much indebted to them. A considerable part of this Lecture is occupied with a parallel between the irritability of vegetable and animal structures, to which we have already alluded; and Hunter seems by this analogy to have been led to espouse strongly the Hallerian doctrine of the independent contractility of muscular fibre. He therefore avoided the error of Whytt by attributing to this source, and not to nervous agency, the actions of the heart and alimentary canal.*

"The great variety of causes of muscular motion, (says Hunter,) make it almost inexplicable; they may be said to be three—the will, passions of the mind, and external stimuli. Those actions arising from the will and the mind appear to be the most simple, because they are totally unintelligible; but those arising from external stimuli are either voluntary or involuntary, for a muscle that acts by command of the will at one time is also capable of being thrown into action by a particular state of mind or external stimulus." (P. 214.)...."The actions of the body that are both voluntary and involuntary are some of the most beautiful circumstances in the machine.

* Hunter's Works; London, 1837, vol. iv. p. 211.

I believe the muscles of respiration are the only perfect instances of it. Fresh air was necessary for our existence, and it was therefore necessary that it should be regulated by some other principle than that of the will; for it is necessary when we sleep and when we *will* the contrary. Therefore our will has its limits of power over the involuntary actions, and the involuntary also have their limits over the actions of the will; each therefore can go only a certain length in opposition to the other." (P. 217.)*

The following summary of Hunter's opinions on associated actions we derive from the chapter on Sympathy, in his Lectures on Surgery. We cannot help thinking that the ideas contained in the last sentence, if fully developed and clothed in modern phraseology, would be found to coincide closely with those which we have expressed in the present article. For our parts, we rejoice to have been anticipated by such a man.

"I attempted to show that the more simple actions arose independently of sensation or of the actions of the nerves; that the nerves, from their specific actions, only become the cause of many actions, but are not the principle of those actions; that, from their termination in the brain, they produce sensations there, from which is formed mind, and that they also give rise to the will, and form the basis of reasoning. I showed that the mind becomes the cause of many involuntary actions in the body, as reason becomes the cause of the voluntary; and that thus the actions of life, of the nerves of the mind, and of the will, arise from impressions being made on each so as to affect their principles." (Vol. i. p. 317.)

It is evident that Hunter had very distinct and accurate ideas of the dependence of the different classes of muscular motions upon the nervous and sensorial powers. He did not attempt, however, to analyze the changes in the nervous system on which these motions are dependent. This was partially done by Sir Gilbert Blane, who took up the subject not long afterwards, with the express intention of extending the results obtained by Hunter; and from his Croonian Lecture on muscular motion, read to the Royal Society in 1788, we shall quote two passages which show how completely he had anticipated various doctrines which have since been propounded as original. After dwelling upon the effects of stimuli applied to the muscular fibre itself, in producing contractions independent of nervous agency, as in the heart and alimentary canal, he proceeds to consider the actions excited by external stimuli, which act remotely, "as in the various instances of sympathy, and in the case of those instincts which nature has implanted for the purpose of self-preservation in brutes, and in the early part of human life." Of the latter he says,† "There is a connexion established between the impression of certain external bodies and the action of certain muscles, analagous to what has already been noticed with regard to the internal motions excited in vessels by the peculiar stimulus of their fluids, nature having instituted certain habitudes between outward stimuli and the moving powers, whereby natural propensities are established equally necessary to the support of life as the internal functions. Thus, in a new born animal, the first contact of the external air excites the act of respiration, and the contact of the nipple excites the act of sucking; both of which actions are absolutely necessary to the maintenance of life, and require the nice cooperation of a great number of muscles,

* Hunter's Works; London, 1837, vol. iv. p. 211.
† Select Dissertations, p. 261.

prior to all experience. Actions of this kind are called instinctive, and differ from voluntary motions in this respect, that the latter are the result of memory and experience, whereas the former are the immediate effect of external impressions, in consequence of an established law of nature, and *independent of consciousness.*" He afterwards remarks that the respiratory movements are the only actions of this class that are constantly performed; and that these, whilst unattended with consciousness in their ordinary exercise, are occasionally under the control of the will. We need scarcely point out the complete correspondence of these views with the opinions which we have already expressed on the subject of instinctive actions.

Sir G. Blane then goes on to investigate how far these movements are dependent on the brain and nerves in animals possessed of these organs:

"There are facts which show that instinctive actions, even in animals endowed with brain and nerves, *do not depend on sensation.* I took a live kitten, a few days old, and divided the spinal marrow, by cutting it across at the neck. The hind paws being then irritated by pricking them, and by touching them with a hot wire, the muscles belonging to the posterior extremities were thrown into contraction, so as to produce the motion of shrinking from the injury. The same effects were perceived in another kitten, after the head was entirely separated from the body. In repeating this experiment, I found that when the spinal marrow was cut through, between the lumbar vertebræ and os sacrum, the posterior extremities lost their irritability, but the part below it, the tail, retained it. It might, therefore, be said that the spinal marrow below the division served as a sensorium; but it may be answered that when the head is cut off its irritability remains, as appears by the motion of the ears when pricked or touched with a hot wire; and as the extremities are also irritable, it will not be said that consciousness and sensation exist in two separated portions of the same body. Nor can it be admitted that sensibility and consciousness may remain in the head after separation; for if mere compression of the carotid arteries abolishes sensation and thought, by interrupting the circulation in the brain, how much more must the superior violence of decapitation have this effect."

Without considering the reasoning in the latter part of this quotation as altogether conclusive, we may point out that Sir G. Blane deduced from his experiments the very same inference which Dr. M. Hall labours to establish; and, as far as the *experiments* of the latter gentleman are concerned, with the same validity; since, as we have already remarked, no *certain* proofs of the absence of sensation can be derived from any source but the human subject. After alluding to the motions of anencephalous monsters as supporting his argument, Sir G. Blane concludes—"These facts show clearly that instinctive, or rather automatic, motions may be excited without the intervention of the sensorium commune, and therefore *without sensation or consciousness.*"

Legallois made a great step in the investigation, by demonstrating the connexion of the sympathetic and excited actions with the spinal cord alone. His work, entitled "Experiences sur le Principe de la Vie," was published in 1812, and consists of Memoirs which had been at different times presented to the Institute and to the Faculty of Medicine in Paris. His great object was to prove that the "principle of life" resides in the spinal cord; that the action of the heart, the respiratory movements, and the contractions of the muscles of the trunk are directly dependent upon it alone; and that it is consequently entitled to rank as a distinct and most important organ, instead of being regarded as a mere bundle of nerves, which seems to have been the estimate formed of it by many of the author's countrymen. By his experiments on the respiratory movements, Legallois showed that the brain itself is not essential to their regular performance; since every part of the cranial contents, except the medulla oblongata, might be removed without interrupting them.* He also proved by experiment that the "life" of every part of the trunk is dependent upon the integrity of its nervous connexions with the spinal cord; and that the integrity of that portion only of the latter organ with which the nerves are connected, is essential to the manifestations of life.† In what these manifestations consist he has explained at the commencement of his work, where he speaks of sensation and motion as the indications of the presence of a living principle. In his zeal to establish the important character of the spinal cord as a distinct organ, Legallois undoubtedly went too far, for we find him maintaining‡ that, when the spinal cord is divided, two centres of volition and sensation are produced: but the results upon which this conclusion was founded are precisely those upon which Blane was led to the inference that motions may be excited through the nervous system without the intervention of sensation; for he found that when the spinal cord of a rabbit was divided below the last dorsal vertebræ, the posterior extremities were agitated when the tail or one of the feet was compressed. In regarding the "principle of sensation and motion" as residing in the spinal cord, and attributing to the brain the function of determining and regulating the actions produced through the medium of the former, we regard Legallois as having greatly erred; but we must not overlook the high value of his researches, both as to the facts which he developed, and the direction of the regard of physiologists towards the unquestionably distinct powers of the spinal cord.

We think it not improbable that many persons who have devoted much attention to *mental* phenomena alone may have arrived at conclusions similar to those which physiologists were attaining by experimental research. No one is likely to arrive at accurate conclusions on subjects like the present who does not add to observation and experiment more or less of acquaintance with the powers and operations of the mind; and it will frequently occur that the results of the two distinct kinds of investigation, physiological and psychological, will confirm or check one another. We can point to one author, at least, on the latter department, whose conclusions will, we think, interest those of our readers who agree with us in the advantage of an extended system of enquiry on topics like the present. The writer of the article Mental Philosophy§ in Rees's Cyclopædia, (published in 1814,) inferred from the consideration of psychical phenomena only, that many actions, both those constantly occurring in the system, and those at first voluntary but subsequently habitual, might be excited through the *sensorial organs* without *sensation* being necessarily produced. He carefully distinguished the *sensorial changes* through which sensation is produced in the mind, from the sensation itself; and argues at some length to prove that in many instances motions are excited by these sensorial changes produced in obedience to an external impression, the mind being unconscious of them either through its direc-

* Op. cit. p. 38. † Ib. p. 34. ‡ Ib. p. 61.

§ The Rev. Dr. L. Carpenter.

tion to other objects, or its state of torpor, as in sleep or apoplexy. If this writer had been acquainted with the experiments of Whytt and Legallois, and with the fact that all nerves are primarily connected with the spinal cord, he would probably have perceived that for the *sensorial changes* to which he alludes this organ alone is required.

None of our readers can be ignorant that, about fifteen years ago, a great advance was made in nervous physiology by the experimental researches of Bell and Mayo in this country, and those of Magendie, Flourens, Fodera, and others on the continent. The results of Magendie's experiments confirmed and extended the views which had been stated by Bell many years before; and in the same manner, the researches of Flourens were little more than a repetition of those of Rolando, also of older date, but little known beyond the country where they were published. The anatomical distinctness of the motor and sensory fibres in the nervous trunks necessarily led to the inference which had been previously supported on other grounds, that the automatic and sympathetic muscular movements can only be produced by the transmission of the external impression to the central organs, and the propagation to the circumference of a motive stimulus. The researches of Rolando, Flourens, Magendie, and other vivisectors, to which we have already alluded, limited the parts of the nervous system necessary to the performance of these instinctive actions to the spinal cord and its cranial prolongations; and the general results set forth by Flourens appear to us to contain nearly all that Dr. M. Hall can be said to have demonstrated. Flourens clearly separated the *excitability* of the nervous fibre, (namely, that power of receiving impressions and of giving rise to functional changes which Bichât included under the general term of *sensibility*,) from true sensibility and voluntary action.* The former he considered as appertaining to the spinal cord and nerves, regarding the nervous trunks as the *excitors* of sensation on one side and of motion on the other, and the spinal cord as the organ by which the *irritations are generalized*, and by which all those sympathetic actions are performed which result from a *transmission of irritation*. He distinctly states that it is in the brain alone that the consciousness of these changes is produced, but that this organ is not concerned in their performance. Not being aware of the distinctness of the sensory and motor fibrils of the nerves, he could not so accurately refer to the transmission of stimuli from the circumference and their reflexion from the centre, as physiologists have subsequently been enabled to do; but we maintain that the knowledge of the facts which he brought forward, when refined and specialised by the splendid discovery of Bell, could not lead to any other conclusion. It is easy to explain the want of attention which the inferences of Flourens himself received in this country. The conclusions drawn from his experiments by Cuvier and his adjuncts in their Report to the Academy, as to the permanence or extinction of sensibility, were precisely opposite to those of their author; for the phenomena which led Flourens to deny the sensibility of the spinal cord, and to point out its excitability (more properly *impressibility*) as a distinct faculty, were regarded by them as proving the persistence of sensibility in that organ,

* Recherches Expérimentales sur le Système Nerveux. Paris, 1824.

and the independence of the cerebral hemispheres regarding this function, except so far as the special sensations are concerned. Founded upon this Report is the statement of Dr. Alison (*Physiology*, p. 131,) that "it is now satisfactorily ascertained that no part of the brain, higher than the corpora quadrigemina, nor of the cerebellum, is essentially concerned in sensation;" or in other words, "that we are to attribute to the spinal cord, and the nerves arising from it, all the physical conditions that are necessary in order that sensation may be felt, and that voluntary efforts may excite muscular contraction." These opinions are now, we think, shown to be invalid by the experiments and pathological observations which we have detailed; and we cannot longer refuse to Flourens the credit of having made a most decided advance in this branch of nervous physiology.

In Dr. Alison's paper on Sympathy* published in 1826, will be found a very full statement of the views of Whytt as modified and rendered more precise by the discoveries of Bell; the object of this profound and sagacious writer was to show the fallacy of the explanation of the respiratory motions propounded by the latter; and to prove that none of the associated actions can be explained by structural connexions between the origins of the nerves. This essay may be looked upon as presenting a fair view of the questions it embraces by one who refused to admit the inferences of Flourens; and we find it distinctly propounded that the sympathetic actions in general are excited by sensations whose influence is *reflected* downwards to parts frequently distant from those on which the stimulating impression is made. Although the writer speaks of the *brain* as connected with them, he was evidently aware that only that portion of the encephalon is essentially concerned which is now usually regarded as the cranial prolongation of the spinal cord.†

We have shown, then, that at this period (about twelve years ago,) the doctrine of the excitement of associated or sympathetic movements by external stimuli acting through the nervous circle of which the spinal cord (to the exclusion of the brain proper) is the centre, was very well understood; that it was also known from the experiments of Legallois that any single division of the spinal cord would act as the centre to the portions of the body connected with it by nervous trunks; and that Dr. Carpenter and Flourens had maintained (the one on psychological, the other on physiological, grounds,) that for this excitement *sensation* is not essential, but rather that series of changes which are antecedent to sensation and which, in the usual state of the system, give rise to it.

We are now, therefore, advanced sufficiently far in our survey to enter upon the claims of the authors of the present time; and we shall briefly consider those which Mr. Mayo has set forth in the pamphlet before us, of which the first portion alone is connected with the subject under discussion. The following are his reasons for thus presenting himself to the public.

"I find that in my former writings in which I have probably too much studied conciseness, I have failed in stating my opinions upon the points referred to so clearly as to prevent their being misunderstood. I have therefore determined to restate them

* Trans. of Edinb. Medico-Chirurgical Society, vol. iii.

† See British and Foreign Med. Review, Vol. III. p. 580.

in a more conspicuous manner and apart from the enquiries with which in my former works they are mixed up. I may mention that I should probably have hesitated to intrude a recapitulation of the following views of these subjects upon the physiological reader, but that I believe them to be susceptible of various practical applications and to form the basis of that true theory of the nervous system which several sensible men in my profession have recently told me they anticipate will be discovered before long." (Pp. 3-4.)

Mr. Mayo attributes to Magendie the honour of the discovery, that the part of the medulla oblongata, corresponding with the root of the fifth nerve, is essential to the perception of all sensations but those of sight, and then gives us his own "larger view" and "juster conclusion," in the following words:

"I believe the segment of the medulla oblongata in which the fifth nerves arise, (or, as I have here more closely phrased it the ROOT of those nerves,) to exert an influence not only *downwards* along the spinal chord, but *upwards*, likewise, towards the brain. I believe that the participation of every part of the nervous system in consciousness depends upon its continuity by nervous substance with that segment. I believe that the organs in which thought and reflection are seated, namely, the cerebrum and cerebellum, are as much dependent for the continuance of their functions on their continuity with the upper part of the medulla oblongata, as are those of the organs of sensation and volition which exist in the spinal chord." (P. 13.)

A little further on, Mr. Mayo adds the following as the "logical deduction" from the facts he has adduced, and others parallel to them, "that the spinal chord has no part in consciousness, and that the cerebrum and cerebellum are equally excluded from their part in consciousness, when they are separated from the medulla oblongata," (p. 16.) We confess that we cannot understand how Mr. Mayo can reconcile the *existence of organs of sensation and volition in the spinal chord*, with its non-possession of *consciousness*. We do not imagine that the spinal cord is possessed of sensibility, or that an animal is conscious of any impressions confined to it; nor, on the other hand, do we believe that although sensibility is a property of cerebral matter, it can be called into exercise, and a sensation produced, by any other means than an impression propagated along the nervous trunks. The humours of the eye will form an image upon a piece of paper as well as upon the retina, but no vision is produced unless the latter be perfect; and the retina cannot conduct to the sensorium any visual impression except that formed by the organ appropriated to it.

Mr. Mayo claims for himself the first enunciation (in his Anatomical and Physiological Commentaries, Part ii., 1823,) of the principle that "an influence may be propagated from the sentient nerves of a part to their correspondent nerves of motion, through the intervention of that part alone of the central organs of the nervous system to which they are mutually attached." But we have seen that the same principle, making allowance for the then prevailing ignorance as to the anatomical distinctness of the motor and sensory fibrils, had been distinctly laid down by Legallois many years before. He also claims the announcement in the same publication, of the idea of the "reflex function," which he allows to Dr. M. Hall the credit of having "followed out with great diligence." We leave the adjustment of this claim, and also the appreciation of its importance to the readers who have attentively followed us in the preceding investigation.

In an addendum to the last edition of his Physiology, Mr. Mayo speaks of having "carefully distinguished" the influence directly "propagated from the sentient nerves of a part to their correspondent nerves of motion," from the "agency of sensation and volition." Now, after careful examination, we can nowhere find that he has gone further than the hypothesis which he advanced in his Anatomical Commentaries, viz. that the connexion between *sentient* surfaces and the action of voluntary muscles may produce motion independently of the *will;* and that the influence independent of will, which occasionally throws voluntary muscles into action, may possibly regulate the unconscious movements connected with the emotions, &c. This hypothesis may have been new to Mr. Mayo's own mind, yet we cannot perceive anything in it which had not been previously advanced; and there certainly is nothing in his statement of it implying that he considered *sensation* as well as volition to be unconcerned in the movements in question. Even this limited view, however, he subsequently relinquishes; and returns to his former opinion, that all instinctive actions, and even those usually termed automatic, are effected by a voluntary impulse. Thus, with regard to the movements of the pharyngeal muscles in deglutition, he "prefers to think" that "by an instinctive law, a voluntary impulse is issued along" the motor nerves in obedience to the *sensation* produced in the *medulla oblongata;* rather than to suppose that volition regulates some movements, and a different principle others, (pp. 21-23.) Certainly Mr. Mayo's notion of volition must be rather more comprehensive than that usually entertained.

It is a little singular that Mr. Mayo should quote Whytt as a supporter of the doctrine that instinctive actions (in which he includes automatic also) are voluntary; for though Whytt attributes them to the *sentient principle*, he carefully distinguishes this from the *rational* principle of the soul, by which alone, according to him, voluntary movements are excited. In the passage which Mr. M. has quoted from the Essay on Vital and other *Involuntary* Motions, as "ringing like true metal," the discerning reader will perceive that Whytt introduces the case of certain undoubtedly voluntary motions, which by habit are performed without a conscious effort of the will, as illustrating, by way of analogy only, the unconscious action of the sentient principle of the mind in producing the movements connected with the organic functions. This error on Mr. Mayo's part, appears to have arisen from an imperfect examination of the whole of Whytt's essay, and the want of due allowance for his peculiar phraseology of which *his own* explanation should be taken. Mr. M.'s present opinion is thus expressed: "Each segment of the double chord, with the nerves arising from it, contains the entire mechanism of sensation, instinct, and volition, although these endowments in the otherwise perfect state of the segment are not manifested unless it be in continuity with the medulla oblongata." (P. 27.) Allowing for the obscurity of this passage—the obvious import of which seems to us to be that in the ordinary state of the spinal cord, the medulla oblongata is the channel through which the endowments of "sensation, instinct, and volition" are manifested, and that when disconnected with it the distinct segments do not manifest them at all, but yet are in possession of them,—we cannot see any essential difference between the idea which Mr. Mayo probably intends to convey, and the inferences drawn by Legallois nearly thirty years ago, to which we have already alluded.

In thus freely criticising the views and opinions, and claims of Mr. Mayo, we are influenced only by one motive, the love of truth; and if we are proved to be in error, it will give us the utmost pleasure to confess it, and, as far as in us lies, to repair the wrong. None can be more willing than ourselves to allow to him whatever credit is justly due to him. He has undoubtedly contributed much, both by his experimental and anatomical researches, to the establishment of results of the highest importance in the physiology of the nervous system; and would he be content to rest his claims to distinction upon his universally acknowledged merits, we think that he would attain a place in general estimation to which such publications as the present are not likely to elevate him.

We shall next advert to the paper of Professor Müller on the "Reflexion of Motions after Perception,"* in which he amplifies the views which he had previously expressed on this subject in his Handbuch der Physiologie. As far as these are concerned we must still maintain the appreciation which we have formerly expressed,† that they contain no opinion which had not been long entertained in this country, being, in fact, only the doctrines of Whytt, with the modifications necessarily resulting from the discoveries of Legallois, Bell, Flourens, &c. ingrafted upon them. That this learned physiologist should have himself regarded them as novel we can only understand by supposing that the opinions of Tiedemann, respecting the dependence of associated movements on the sympathetic nerve were then prevalent in Germany. Now Dr. Alison had, many years previously to Professor Müller, laid down very clearly the distinction between the *organic sympathies*, which he refers to the *great sympathetic*, and the *sympathetic movements*, which he justly regarded as dependent upon the cerebro-spinal axis. The experimental researches of Brâchet, too, led to the same conclusion; and indeed this physiologist made out a distinct case of *reflex action without sensation*, in maintaining that the motions of the parietes of the stomach depend upon an impression conveyed to the medulla oblongata by the par vagum, and transmitted down again in the form of a motive impulse. Although not according with Dr. M. Hall in several points, Professor Müller seems disposed to admit that *sensation* is not an essential link in the chain of reflected actions; for he observes‡ that " the reflected motions on stimuli of the skin, which take place after the removal of the brain, do not contain any proof that the stimulus excited true sensation in the spinal marrow; it is rather the centripetal conduction of the nervous principle which commonly takes place in sensations, but which here is no longer sensation, because it is no longer conducted to the brain, the organ of consciousness. During health, also, numerous reflected motions result from stimuli of the skin, which do not come as true sensations to the consciousness, but still may excite violent impressions on the spinal marrow." He also argues in

* Philosophical Magazine, vol. x.

† British and Foreign Medical Review, vol. iii. p. 581.

‡ Op. cit. p. 188.

favour of an opinion which we have taken some pains to uphold, that involuntary movements may arise from sensations (particularly those of a special nature,) as directly as those characterized as reflex or excito-motory by Dr. Hall from impressions only.

To those who have followed us through the preceding retrospect, we think it will appear that the following opinions had been maintained by different authors previously to the publication of the researches of Dr. Hall and Professor Müller: 1. That the sympathetic or associated movements are involuntarily excited by a stimulus conveyed to the central parts of the cerebro-spinal nervous system along one portion of the nervous trunks, and centrifugally transmitted by another. 2. That for the reflexion of this stimulus, the brain is not necessary, but the spinal cord and its nerves alone. 3. That any segment of the spinal cord may act independently of the rest, so far as the parts of the body are concerned, with which it possesses nervous communications. 4. That sensation is not an essential link in the chain of processes, this mental condition being a result of those organic changes which are excited by the external impression, and which propagate its influence to the muscles.

We do not mean to assert, however, that any one physiologist maintained all these doctrines, in the form in which we have expressed them; although Flourens undoubtedly approached so near to them, that, if he had been acquainted with the previous investigations of Bell, we cannot but think that he would have been led to see their bearing upon his own conclusions. Now, it appears to us that it is in the harmonious combination of these doctrines into a uniform system, and the application of it to the explanation of many phenomena which were not formerly regarded as explicable on such principles, that Dr. Hall's great merit consists, and not to anything particularly novel in the opinions themselves. The claims to *discovery* on a question like the present are very difficult to adjust, if all the vague thoughts which have preceded them respecting the subject they involve are allowed to be brought in evidence of previous right;—thoughts, too, which would perhaps have been forgotten, if the clear statements of the writer whose claims to originality are opposed had not revived them. Admitting that this is in some degree the case with regard to Dr. Hall, we still think that the clear statements of the previous authors we have quoted will, if fairly examined, bear out the opinion we have now given. We have been pleased to find that so able and intelligent a writer as Mr. Owen has come to precisely the same conclusions with ourselves as to the validity of Dr. Hall's opinions and the justice of his claims.

"The essential character of the actions of the brain, whether as a recipient or transmitter of impressions, is consciousness of the action: but this property of consciousness is not possessed by the spinal cord; probably not by the medulla oblongata. Whenever, therefore, an impression received by an excitable nervous fibre is transmitted through the unconscious part of the central axis to the exciting fibre of muscular motion, the latter phenomenon is unaccompanied with consciousness or sensation. That the spinal cord, or any segment of it, possesses the power of transmitting an impression from an excitable to an exciting nervous fibre, has been known and admitted as a fundamental fact in physiology since the experiments of Whytt, Blane, and Mayo. To the latter physiologist we are more especially indebted for the most decisive experiments in proof, and the clearest enunciation, of this property of the central nervous axis. Recently the automatic animal motions resulting from this property have been grouped together more *extensively than had before been done, and the morbid phenomena ably traced out by Dr. M. Hall.*"*

Dr. Hall claims, however, much more than this: he thinks that he has succeeded in demonstrating the existence of a "System of Excito-motory Nerves, *physiologically* distinct from the nerves of sensation and voluntary motion." It is a little remarkable, however, that those writers who were disposed to accord with his views in other respects, have, in almost every instance, expressed their conviction that this supposition is unphilosophical, because unnecessary; and that there is no reason to suppose that the very same fibrils may not conduct those impressions to the spinal cord, which, acting through it alone give rise directly to motive influence, or, propagated to the brain, cause sensations to be formed in the mind. This was the opinion of Professor Müller,† Mr. Carpenter,‡ Dr. J. Reid,§ of Mr. Owen,|| and others. We shall quote the remarks of the last, as giving, in our opinion, a very fair statement of the question.

"There is not a single phenomenon of automatic motion in parts supplied by spinal nerves which may not be accounted for on the demonstrated property of the central axes to transmit impressions from the excitable to the exciting nerves, at any part where they are connected to it, independently of the rest; and I am at a loss to understand why impressions, so received by the spinal cord, should not also be transmitted by the brain, (its continuity with that organ being uninterrupted,) without the necessity of supposing a class of nervous fibres for conveying, in this case, impressions to the spinal cord, distinct from a second class which are supposed to transmit to the motive fibres those impressions which are not afterwards propagated to the brain."

Mr. Owen shews himself, however, quite willing to modify his opinions on the subject, if called upon to do so by new facts; for he says, at the same time, "It remains to be seen whether anatomy will establish the existence of these four classes of nervous fibres, which, so far as I can understand Dr. Hall's hypothesis of the reflex function, are called in to account for the voluntary and automatic muscular motions."

This question we cannot regard as by any means decided by the investigations of Mr. Grainger. Regarding the anatomy of the spinal cord this gentleman has not, as we have shewn, arrived at any peculiarly novel results; and, without calling in question a single one of his facts, we must take leave to withhold our assent to the theory erected upon them. Not because any direct and certain evidence can be produced against it, however, do we regard it as unstable, but because we cannot discern either solidity in its foundation, or firmness and connexion in the superstructure. It is to be remembered that the doctrine of the relative functions of the grey and white matter is by no means entitled to rank as a physiological certainty; and, however ingenious may be the deductions drawn from it, they must be looked upon with great suspicion, unless they meet with substantial support elsewhere. We may take this opportunity of expressing our high appreciation of the general character of Mr. Grainger's work, from which it will be evident that we have drawn much valuable

* Hunter's Works, vol. iv. p. 202, note.
† Op. cit.
‡ Edinburgh Med. and Surg. Journal, vol. xlviii. p. 43. § Ditto, vol. xlix.
|| Loc. cit.

information; and, although we differ from him in several important physiological views, and in the estimation we attach to the labours of Dr. Hall, we willingly bear testimony to the very candid and discriminating spirit which generally pervades his book.

We have but a few words more to say of Dr. Hall. His want of accurate acquaintance with the opinions of older physiologists, to which we have already alluded, has led him to regard many of his doctrines as novel which are very far from being so. To some of his pathological explanations we have alluded in a former article,* and have shewn that they differ only in words from those commonly given. With regard to the function of respiration, again, the comprehension of which within his excito-motor system he regards as a great advance in the enquiry, he seems quite unaware that, before the time of Bell, it was universally considered as on a par with other associated movements. Whytt states this as clearly as possible, and describes the action of the stimulus originating in the lungs, and transmitted to the muscles through the sensations produced in the brain, (or rather, to use his own language, through the excitement of the *sentient principle*, not always implying *sensation*.) The great pains which Bell took to set it in a different light produced a corresponding effect on the minds of many physiologists, particularly in England; but his *respiratory system* was never received in an unqualified manner in any but English schools; and, as we have already seen, the question was early placed on its true footing by Dr. Alison. The introduction of the function of respiration by Dr. M. Hall, therefore, into his excito-motor system, was merely to restore it to its true affinity with those other associated movements from which (following the doctrine of Bell) he had at first unjustly separated it.

With one more observation we shall conclude our references to Dr. Hall's doctrines and claims. He imagines that to him is due the establishment of the fact that the nervous influence may operate in directions not only incident and reflex, but also direct and retrograde, or, in other words, downwards and upwards, in regard to the spinal marrow. Now, this is saying nothing more than that a stimulus affecting one part of the body may produce motions in other parts whose nerves arise either above or below the insertion of those which convey the impression to the spinal cord. This was a fact known to every one long before Dr. Hall's time; and, although in the view of those who regarded the spinal cord as a mere bundle of cerebral nerves it might be a matter of astonishment that such a retrograde action could occur, no physiologist who considered it an independent organ would have more difficulty in accounting for the fact that a stimulus conveyed to the lumbar region might excite the motor nerves of the cervical portion, or that a stimulus incident on the dorsal region might excite both cervical and lumbar motor nerves, (in the first of which cases the action would be *retrograde*, according to Dr. Hall, in the second both *retrograde* and *direct*,) than that a stimulus affecting the cervical portion of the cord should be conveyed (*directly*, according to Dr. Hall,) to the motor nerves of the back or loins.†

† A passage in one of Dr. M. Hall's recent Lectures (*Lancet*, February 17, p. 729,) induces us to refer to one topic, which we should not otherwise have thought it neces-

In taking our leave of Dr. Hall, we would assure him and his partisans that we have been actuated by no motive, in the examination into his claims which we have thought it our duty to make, save an honest desire to arrive at the truth, and to set their investigations on their proper footing; and we would remark, that we are quite sure that these claims would have received more attention in various quarters had they been more moderate and discriminative.

---

